# Tri-1,3,4-Oxadiazoles Modified with Nitroimine: Balancing Energy, Sensitivity, and Thermal Stability

**DOI:** 10.3390/molecules30214224

**Published:** 2025-10-29

**Authors:** Fangming Chen, Qiong Yu, Lei Li, Kejia Peng, Chenguang Zhu, Wenbin Yi

**Affiliations:** School of Chemistry and Chemical Engineering, Nanjing University of Science and Technology, Nanjing 210094, China

**Keywords:** 1,3,4-oxadiazole, energetic materials, insensitivity, thermal stability

## Abstract

Achieving a balance of good thermal stability, high performance, and appropriate sensitivity in materials remains a primary research goal in energetic materials. In this study, a series of dinitrimine-functionalized tris-1,3,4-oxadiazole-based energetic compounds was synthesized. Dinitroimmine **5** was found to possess favorable thermal stability (*T*_d_ = 180 °C), superior mechanical sensitivity (IS = 25 J, FS = 240 N), and good detonation velocity (*v*_D_ = 8372 m s^−1^). These results suggest that this polyheterocyclic backbone structure facilitates the synthesis of high-performance energetic compounds with application potentials.

## 1. Introduction

Over the past few decades, significant research interest has focused on energetic materials, leading to the synthesis of numerous high-energy density materials (HEDMs) [1,2,3,4,5,6]. Mechanical and thermal stability are critical properties of HEDMs that ensure safety during storage, transportation, and utilization. Within this field, compounds with high energy but low sensitivity—specifically those with impact sensitivity lower than that of TNT (IS = 15 J)—are a key area of research [7,8]. Notably, only materials with decomposition temperatures exceeding 150 °C are considered suitable for practical use [9,10].

**Figure 1:**
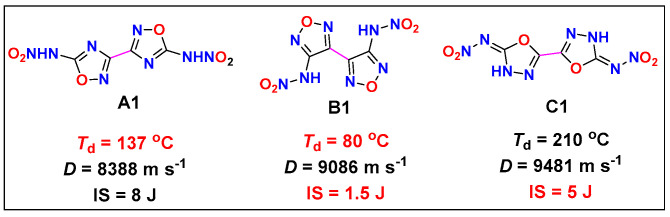
Properties of nitroamino/nitroimmine-substituted bis-oxadiazoles.

Nitrogen-rich heterocycles have garnered considerable attention due to their high heat of formation, high densities, and clean gaseous combustion products [11]. Among these, oxadiazole exists in three stable isomers: 1,2,5-oxadiazole, 1,2,4-oxadiazole, and 1,3,4-oxadiazole [12]. Of particular interest are oxadiazole-based compounds due to their high nitrogen and oxygen content, which contributes to their energetic properties [13,14]. Energetic nitramine derivatives of oxadiazole often exhibit impressive detonation velocities (Figure 1); for example, compounds A1 [15], B1 [16], and C1 [17] demonstrate detonation velocities of 8388 m s^−1^, 9086 m s^−1^, and 9481 m s^−1^, respectively. However, the inherent low bond dissociation energy (BDE) of the N–NO_2_ bond typically results in poor stability for such compounds [18,19,20]. For example, **A1** exhibits an impact sensitivity of IS = 8 J and a decomposition temperature of 137 °C, while **B1** shows even lower values with IS = 1.5 J and *T*_d_ = 80 °C. Notably, compound **C1** (*T*_d_ = 210 °C) stands out as an exception, showcasing good thermal stability due to the tautomerization of its 1,3,4-oxadiazole nitroimine structure. Despite this, **C1**’s high impact sensitivity limits its practical applications.

**Figure 2:**
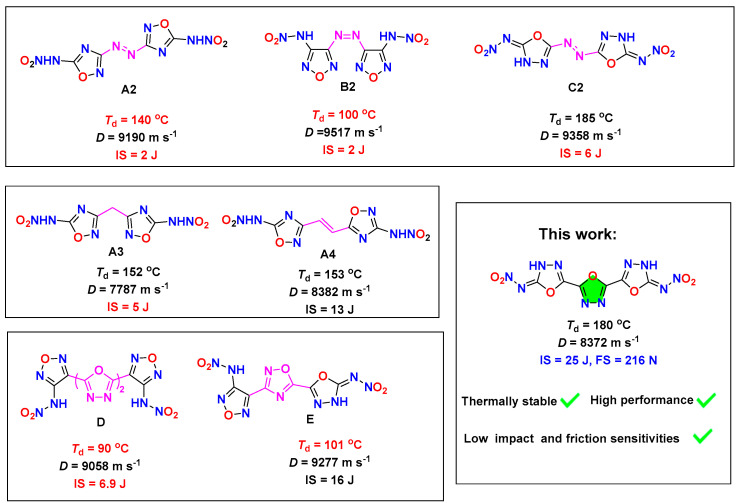
Properties of nitramino/nitrimmino-substituted bis-oxadiazoles bridged by different fragments.

To enhance the performance of energetic materials, introducing bridges—such as azo, methyl, vinyl, or nitrogen-rich heterocycles—between energetic units has emerged as a common structural modification strategy [21,22,23]. Compared to **A1**–**C1**, compounds **A2**–**C2** bridged by an azo group exhibit significantly improved detonation properties (**A2**, *v*_D_ = 9190 m s^−1^; **B2**, *v*_D_ = 9517 m s^−1^; **C2**, *v*_D_ = 9358 m s^−1^), as shown in Figure 2. However, these azo-bridged compounds also demonstrate poor impact sensitivity (e.g., **A2**, IS = 2 J; **B2**, IS = 2 J; **C2**, IS = 6 J) [24,25,26]. In contrast, methyl and vinyl bridged compounds (**A3** and **A4**) demonstrate enhanced thermal stability (**A3**, *T*_d_ = 152 °C; **A4**, *T*_d_ = 153 °C), albeit with lower detonation characteristics (e.g., **A3**, *v*_D_ = 7787 m s^−1^; **A4**, *v*_D_ = 8382 m s^−1^) [27,28]. Furthermore, oxadiazole-bridged compounds **D** and **E** exhibit significantly improved mechanical stability, with impact sensitivity values of IS = 6.9 J and IS = 16 J, respectively [29,30].

In order to achieve a balance between good thermal stability, insensitivity, and high energy, the present study combines the 1,3,4-oxadiazole nitrimine and oxadiazole bridge to develop a series of dinitramine-functionalized tris-1,3,4-oxadiazole-based energetic compounds. All synthesized compounds underwent comprehensive characterization using techniques such as IR, ^1^H NMR, and ^13^C NMR. The crystal structure of compound **6** was confirmed via X-ray diffraction. Notably, compounds **5** and **7** exhibit promising applications in explosive materials, with **5** showing potential as an explosive and **7** demonstrating suitability as a primary explosive. This research contributes to the development of energetic materials by addressing key challenges in balancing thermal stability, insensitivity, and high-energy density.

## 2. Results and Discussion

### 2.1. Synthesis

**Scheme 1:**
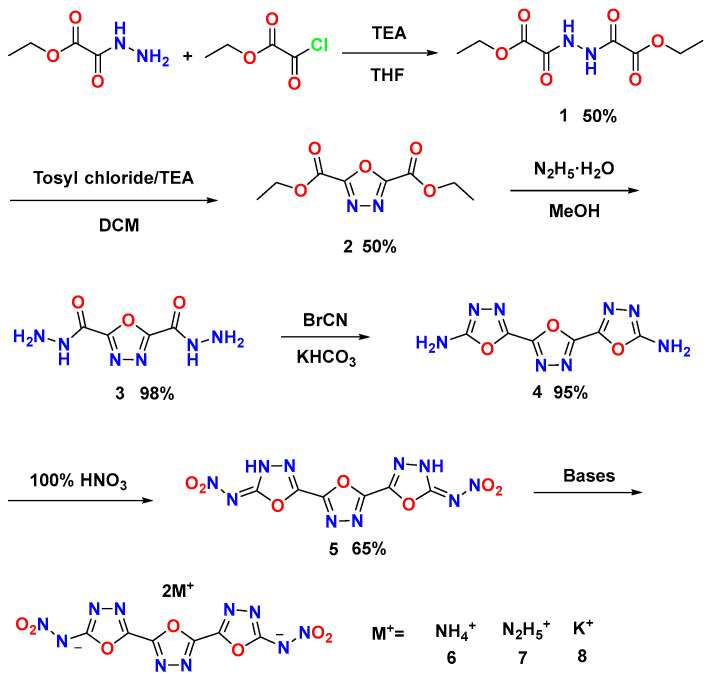
Synthesis of compounds **2**–**8**.

As shown in Scheme 1, a mixture of monoethyl oxalate hydrazide and ethyl oxalyl chloride was reacted in tetrahydrofuran in the presence of triethylamine (TEA) to afford diethyl 2,2′-(hydrazine-1,2-diyl) bis(2-oxoacetate) (**1**). Compound **1** was then treated with triethylamine (TEA) and *p*-toluenesulfonyl chloride (TsCl) in dichloromethane, resulting in the formation of compound **2**. Subsequent treatment of 1,3,4-oxadiazolederivative **2** with hydrazine monohydrate in methanol afforded 1,3,4-oxadiazole-2,5-dicarbohydrazide (**3**). A solution of **3** in methanol/water was cooled to 0 °C, followed by sequential addition of cyanogen bromide and potassium bicarbonate with stirring. The reaction mixture was maintained at 25 °C for 3 h, forming compound **4**. The target compound, 2,5-bis-(2-amino-1,3,4-oxadiazol-5-yl)-1,3,4-oxadiazole (**5**), was then synthesized via nitration of **4** using 100% HNO_3_. Further treatment of **5** with ammonia in ethanol, hydrazine monohydrate or potassium hydroxide produces ammonium salt (**6**), hydrazinium salt (**7**) or potassium salt (**8**), respectively. The attempt to obtain hydroxylaminium salt failed.

### 2.2. X-Ray Diffraction

**Figure 3:**
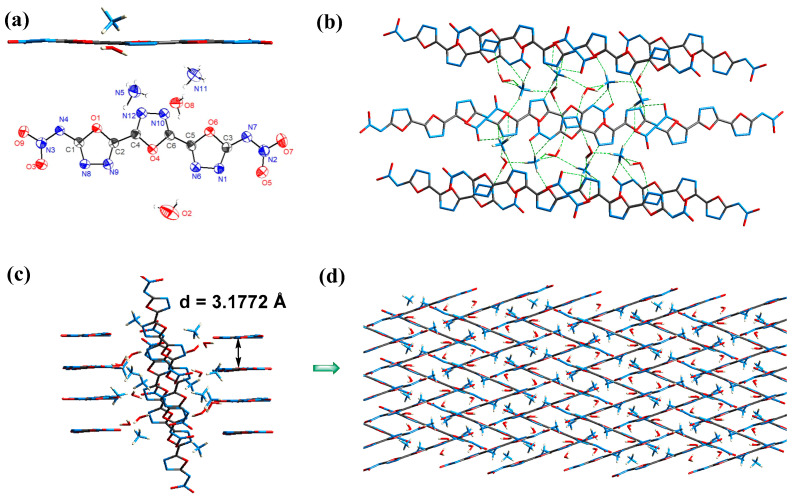
(**a**) Molecular structure of crystal **6**·2H_2_O. (**b**) Crystal packing projection of compound **6**·2H_2_O along the *a*-axis with HBs represented by green dotted lines. (**c**) Layer-by-layer diagram of **6**·2H_2_O along the *b*-axis. (**d**) Cross-packing diagram of **6**·2H_2_O along the *c*-axis.

Crystal **6**·2H_2_O (CCDC 2087554) has a calculated density of 1.702 g cm^−3^ at 273 K, which crystallizes in the monoclinic space group *P*2_1_/c, with four molecules per unit cell (detailed crystallographic data in Tables S1–S7, ESI). The crystal was obtained by slowly evaporating mixed solvents of methanol and acetonitrile. Its structure reveals near coplanarity of the three C-C-bonded 1,3,4-oxadiazole rings and two N-nitro groups (Figure 3a), supported by key torsional angles: O3-N3-N4-C1 = 1.837°, O9-N3-N4-C1 = −178.457°, O1-C2-C4-N12 = −0.489°, O1-C2-C4-O4 = −179.062°, N10-C6-C5-O6 = 4.102°, N10-C6-C5-N6 = −177.048°, C3-N7-N2-O5 = −3.184°, and C3-N7-N3-O7 = 176.727°. As illustrated in Figure 3b, the crystal structure of **6**·2H_2_O exhibited an optimal three-dimensional packing diagram along the *a*-axis, extending through strong hydrogen bond interactions. Along the *b*-axis, two stacking modes (layer-by-layer and cross-packing) were observed. The distance between the two planes is 3.1772 Å (Figure 3c), which is shorter than the typical geometric parameters of aromatic π-π interactions (3.65–4.00 Å) [31]. The cross-packing diagram of **6**·2H_2_O along the *c*-axis shows a fishing net arrangement (Figure 3d).

### 2.3. Weak Interactions

**Figure 4:**
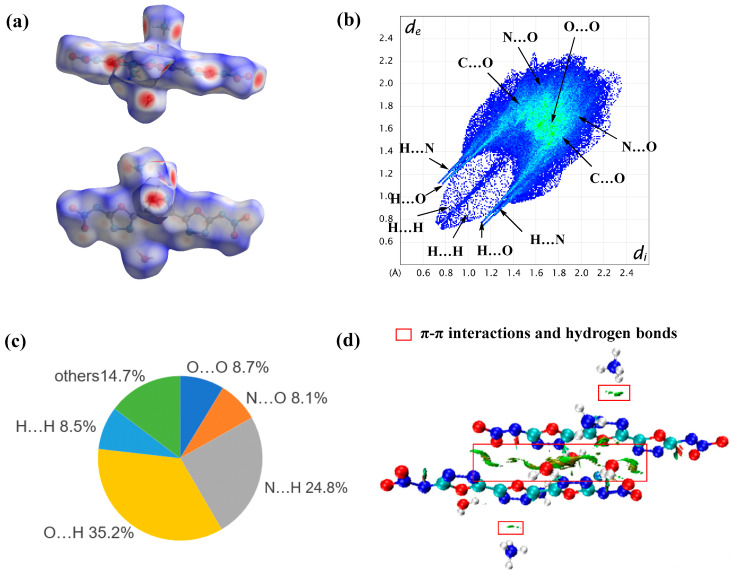
(**a**) Hirshfeld surfaces in crystal stacking for **6**·2H_2_O. (**b**) Two-dimensional fingerprint plot for **6**·2H_2_O. (**c**) The pie graph for **6**·2H_2_O. (**d**) Noncovalent interaction analysis and π-π interactions for **6**·2H_2_O.

**Figure 5:**
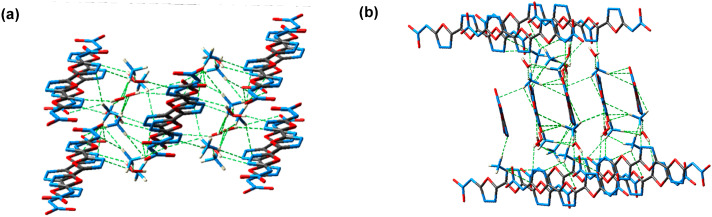
(**a**) Intramolecular and intermolecular hydrogen bonds in **6**·2H_2_O. (**b**) Interlayer hydrogen bonds in **6**·2H_2_O.

The nature of intermolecular interactions within the crystal structures of **6**·2H_2_O was further investigated using Hirshfeld surface analysis and noncovalent interaction (NCI) analysis [32,33]. On the Hirshfeld surfaces, red spots highlight regions of significant close contacts, while blue areas indicate regions of minimal interaction. As shown in Figure 4c, distinct red dots are observed on the side of the Hirshfeld surface, and three pairs of peaks can be identified in the lower left corner of the fingerprint image: O…H/H…O, N…H/H…N, and H…H weak interactions, which account for 35.2%, 24.8%, and 8.5% of total weak interactions, respectively, summing to over 65%. These hydrogen bonds can be categorized into intramolecular and intermolecular hydrogen bonds. As depicted in Figure 5a, the hydrogen bonding interactions in crystal **6**∙2H_2_O are predominantly intermolecular. While intermolecular hydrogen bonds can enhance molecular sensitivity by strengthening lattice strength [34], the interlayer hydrogen bonds present, as shown in Figure 5b, can improve the interlayer sliding ability of molecules, thereby enhancing their resistance to mechanical stimuli [35]. Furthermore, the 3D network structure formed by the dense hydrogen bonds in this crystal can significantly increase the crystal’s lattice energy, thus greatly improving its structural stability [36].

### 2.4. Physicochemical Properties

The physical and energetic properties of new compounds were thoroughly evaluated using a combination of experimental techniques and theoretical calculations. The densities of all compounds were measured by using an Anton Paar gas pycnometer (25 °C). The densities of these compounds were found to be 1.67–2.12 g cm^−3^. Thermal stability was assessed through DSC analysis. As demonstrated in the Supplementary Figures S2–S6, all compounds demonstrate thermal decomposition temperatures exceeding 150 °C. Specifically, the thermal decomposition of compounds **5**–**7** occurs at temperatures ranging from 171.2 to 192 °C. Compounds **4** and **8** have higher thermal decomposition temperatures of 276 °C and 204 °C, respectively.

In order to estimate the energetic performance, the heats of formation (Δ_f_*H*) for compounds **4**–**8** were calculated using Gaussian 16 [37]. The geometric optimization and frequency analyses of compounds were fulfilled using the B3-LYP [38] functional with the 6-31G** [39,40] basis set, and single energy points were calculated at the M06-2X/DEF2-TZVP level. All the optimized structures were characterized to be true local energy minima on the potential-energy surface without imaginary frequencies. Additionally, the sublimation enthalpies of compounds **4** and **5** were calculated using the method reported by Mathieu [41,42]. Based on the calculated Δ_f_*H* values and experimental densities, the use of EXPLO5 successfully achieved the desired detonation velocity (*v*_D_) and pressure (*P*) [43]. As presented in Table 1, compounds **6** and **8** demonstrate superior calculated detonation performance (*v*_D_ = 7817–8076 m s^−1^, *p* = 24.0–25.6 GPa) in comparison to that of TNT [44]. Compound **5** exhibits a performance that is marginally below the calculated detonation performance (*v*_D_ = 8372 m s^−1^, *p* = 29.5 GPa) of RDX [45] (*v*_D_ = 8748 m s^−1^, *p* = 34.9 GPa). It has been demonstrated that compound **7** exhibits superior calculated explosive velocity (*v*_D_ = 8939 m s^−1^) in comparison to RDX and Pd(N_3_)_2_ [46] (*v*_D_ = 5877 m s^−1^). Sensitivity testing was conducted in accordance with standard BAM methods. The impact sensitivity of the compounds (IS = 25 J) under investigation is lower than that of TNT (IS = 15 J), with the exception of compound **7**, which exhibits a higher impact sensitivity (IS = 1.5 J). It has been demonstrated that all compounds exhibit insensitive friction sensitivity (FS = 72 and 240 N).

## 3. Materials and Methods

Experimental section caution: all the nitrogen-rich compounds used are energetic materials and may explode under certain conditions. Appropriate safety precautions should be taken when preparing and/or handling. Only small quantities should be prepared and studied.

### 3.1. Reagents and Instruments

The reagents were purchased from Admas (Emeryville, CA, USA) and Aladdin (Shanghai, China) and used directly without any additional treatment. ^1^H NMR and ^13^C NMR spectra were recorded by Bruker AVANCE III 500 MHz (Bruker, Billerica, MA, USA), operating at 500 and 75 MHz. DMSO-*d_6_* was used as a solvent and for field locking. Chemical shifts are reported relative to (CH_3_)_4_Si in the ^1^H and ^13^C spectra. The decomposition (onset temperature) points were obtained using a differential scanning calorimeter. The decomposition (onset temperature) points were measured using a differential scanning calorimeter (NETZSCH DSC 300 Caliris, NETZSCH, Selb, Germany) at a heating rate of 5 °C min^−1^. Elemental analyses of C/H/N were performed on a Vario Micro cube Elementar Analyser (Elementar, Langenselbold, Germany).

### 3.2. Experimental Methods

**Diethyl 2,2′-(hydrazine-1,2-diyl)-bis(2-oxoacetate) (1):** Ethanedioic acid, monoethyl ester, hydrazide (20 mmol, 2.64 g) was dissolved in 100 mL of tetrahydrofuran solution and cooled to 0 °C in an ice-salt bath. Ethyl oxalyl monochloride (20 mmol, 2.73 g) was added dropwise, and 4 mL of triethylamine was added slowly, brought to room temperature, and stirred overnight. The solvent was evaporated, washed with water, filtered, and dried at room temperature to obtain a white solid (2.32 g, 50% yield). ^1^H NMR (500 MHz, DMSO-*d_6_*) δ 11.05 (s, 1H), 4.29 (q, *J* = 7.1 Hz, 2H), 1.29 (t, *J* = 7.1 Hz, 3H). ^13^C NMR (126 MHz, DMSO-*d_6_*) δ 159.92 (s), 156.29 (s), 62.98 (s), 40.43 (s), 40.27 (s), 40.11–40.10 (m), 40.02 (d, *J* = 21.0 Hz), 39.77 (s), 39.61 (s), 39.52 (d, *J* = 21.0 Hz), 14.23 (s); elemental analysis (%) calcd. for C_8_H_12_N_2_O_6_ (232.19); C 41.38, H 5.21, N 12.07; found, C 41.52, H 5.33, N 12.14.

**Diethyl 1,3,4-oxadiazole-2,5-dicarboxylate (2):** Compound **1** (30 mmol, 7 g) was dissolved in 100 mL of dichloromethane. Then, *p*-Toluenesulfonyl chloride (30 mmol, 5.8 g) and 6 mL of triethylamine (TEA) were added, and after stirring at room temperature for 22 h, the solvent was evaporated, washed with water, filtered, and dried at room temperature to obtain a white solid (3.2 g, 50% yield). ^1^H NMR (500 MHz, CDCl_3_) δ 4.49 (q, *J* = 7.1 Hz, 4H), 1.41 (t, *J* = 7.2 Hz, 6H). ^13^C NMR (126 MHz, CDCl_3_) δ 157.58 (s), 153.57 (s), 64.28 (s), 14.12 (s). IR (KBr pellet): 2989, 1752, 1740, 1537, 1528, 1470, 1447, 1411, 1394, 1368, 1309, 1289, 1229, 1153, 1114, 1030, 1013, 993, 973, 848, 792, 775, 638; elemental analysis (%) calcd. for C_10_H_10_N_2_O_5_ (214.18); C 44.86, H 4.71, N 13.08; found, C 44.80, H 4.65, N 13.18.

**1,3,4-oxadiazole-2,5-dicarbohydrazide (3):** Compound **2** (30 mmol, 6.42 g) was dissolved in 100 mL of methanol and cooled to 0 °C. Then, 85% hydrazine hydrate (90 mmol) in 30 mL of methanol solution was slowly added dropwise. The reaction was carried out at 0 °C for 1 h. After stirring for 3 h at room temperature, the precipitate was filtered and vacuum dried to obtain a yellow solid (98% yield). ^1^H NMR (500 MHz, DMSO-*d_6_*) δ 6.43 (s, 4H). ^13^C NMR (126 MHz, DMSO-*d_6_*) δ 158.57 (s), 151.74 (s). IR (KBr pellet): 3303, 3275, 3180, 3040, 3005, 1756, 1692, 1628, 1598, 1549, 1521, 1509, 1397, 1334, 1308, 1292, 1216, 1173, 1137, 1044, 1007, 973, 962, 949, 881, 867, 790, 765, 727, 675, 632, 560; elemental analysis (%) calcd. for C_4_H_6_N_6_O_3_ (186.05); elemental analysis (%) calcd. for C_4_H_6_N_6_O_3_ (186.13); elemental analysis (%) calcd. for C 25.81, H 3.25, N 45.15; found, C 25.85, H 3.23, N 45.17.

**2,5-Bis-(2-amino-1,3,4-oxadiazol-5-yl)-1,3,4-oxadiazole (4):** Compound **3** (0.93 g, 5 mmol) was dispersed into a mixture of 25 mL of water and 25 mL of solution. Cyanogen bromide (11 mmol, 1.166 g) was then added, and the reaction mixture was stirred at room temperature for 3 h. Subsequently, KHCO_3_ (12 mmol, 1.2 g) was added, and the reaction mixture was filtered, washed with water, and dried in vacuo to an orange-colored solid (1.12 g, 95% yield). ^1^H NMR (500 MHz, DMSO-*d_6_*) δ 7.98 (s, 4H). ^13^C NMR (126 MHz, DMSO-*d_6_*) δ 165.10 (s), 153.08 (s), 146.00 (s); elemental analysis (%) calcd. for C_6_H_4_N_8_O_3_ (236.15); C 30.52, H 1.71, N 47.45; found, C 30.41, H 1.89, N 47.55.

**2,5-Bis-(2-nitroamino-1,3,4-oxadiazol-5-yl)-1,3,4-oxadiazole (5):** A minimal quantity of compound **4** (5 mmol, 1.18 g) was meticulously introduced to 10 mL of 100% nitric acid at 0 °C. The mixture was then subjected to stirring at the same temperature for a duration of 1 h. Thereafter, the temperature was gradually increased to room temperature, and the stirring continued for a period of 12 h. Subsequently, the mixture was splashed into ice, resulting in the precipitation of a white solid. The solid was then filtered and dried under vacuum conditions (1.06 g, 65%). ^1^H NMR (500 MHz, DMSO-*d_6_*) δ 5.79 (s, 2H). ^13^C NMR (126 MHz, DMSO-*d_6_*) δ 163.77 (s), 153.38 (s), 145.72 (s). IR (KBr pellet): 3231, 1618, 1578, 1524, 1494, 1386, 1353, 1294, 1244, 1167, 1141, 1104, 1069, 1047, 1034, 1005, 970, 959, 947, 840, 777, 715, 695, 659, 562; elemental analysis (%) calcd. for C_6_H_2_N_10_O_7_ (326.15); C 22.10, H 0.62, N 42.95; found, C 22.23, H 0.69, N 42.87.

**General procedures for salts 6–8:** Compound **5** (326 mg, 1 mmol) was dissolved in 5 mL of methanol at 0 °C, and 2 M ammonia–ethanol solution, hydrazine hydrate (85%) in methanol, or aqueous potassium hydroxide was added slowly and dropwise to the pH of the reaction solution until it was neutral. The mixture was then filtered to precipitate and dried under vacuum conditions. (**6**, 0.34 g, yield 95.0%; **7**, 0.37 g, yield 85.0%; **8**, 0.38, 95%).

**Diammonium 2,5-Bis-(2-nitroamino-1,3,4-oxadiazol-5-yl)-1,3,4-oxadiazole (6):** ^1^H NMR (500 MHz, DMSO-*d_6_*) δ 7.13 (s, 8H). ^13^C NMR (126 MHz, DMSO-*d_6_*) δ 166.95 (s), 153.62 (s), 147.32 (s). IR (KBr pellet): 3501, 3169, 1631, 1515, 1487, 1402, 1315, 1270, 1249, 1171, 1142, 1074, 1026, 1010, 957, 951, 850, 779, 745, 723; elemental analysis (%) calcd. for C_6_H_8_N_12_O_7_ (360.06); C 20.01, H 2.24, N 46.66; found, C 20.12, H 2.44, N 46.73.

**Dihydrazinium 2,5-Bis-(2-nitroamino-1,3,4-oxadiazol-5-yl)-1,3,4-oxadiazole (7):** ^1^H NMR (500 MHz, DMSO-*d_6_*) δ 7.99 (s, 10H). ^13^C NMR (126 MHz, DMSO-*d_6_*) δ 166.96 (s), 153.66 (s), 147.35 (s). IR (KBr pellet): 3198, 1621, 1509, 1485, 1406, 1333, 1307, 1259, 1167, 1136, 1077, 1028, 952, 852, 823, 777 756, 744, 722, 666; elemental analysis (%) calcd. for C_6_H_10_N_12_O_7_ (360.06); C 18.47, H 2.58, N 50.25; found, C 18.35, H 2.49, N 50.37.

**Dipotassium 2,5-Bis-(2-nitroamino-1,3,4-oxadiazol-5-yl)-1,3,4-oxadiazole (8):** ^13^C NMR (126 MHz, DMSO-*d_6_*) δ 166.97 (s), 153.65 (s), 147.36 (s). IR (KBr pellet): 3233, 1611, 1511, 1482, 1409, 1276, 1202, 1128, 1076, 1027, 993, 953, 911, 855, 778, 755, 737, 721, 665; elemental analysis (%) calcd. for C_6_N_10_O_7_K_2_ (401.92); C 17.91, H 0, N 34.82; found, C 17.83, H 0.21, N 34.93.

## 4. Conclusions

In summary, a series of tris-oxadiazole-based nitramine-containing energetic compounds has been synthesized and characterized. The molecular planarity phenomenon has been explained using single-crystal X-ray diffraction. Compared to oxadiazole nitramides that have already been synthesized, neutral compound **5** demonstrates an optimal balance of high energy (*v*_D_ = 8372 m s^−1^), minimal sensitivity (IS = 25 J) and an ideal thermal decomposition temperature (*T*_d_ = 180 °C). The dihydrazinium salt **7** has a calculated explosive velocity of 8939 m s^−1^, a thermal decomposition temperature of 171.2 °C and an impact susceptibility of 1.5 J. Its high explosive performance, good thermal stability and suitable susceptibility make it a promising candidate for use as a green primary explosive. Clearly, the synthetic strategy and the balance of properties of this tricyclic skeleton are instructive for designing high-energy, low-sensitivity energetic compounds.

## Figures and Tables

**Table 1:** Properties of energetic compounds.

Comp.	*T*_d_ ^a^ (°C)	*ρ*^ b^ (g cm^−3^)	Δ_f_*H* ^c^ (kJ mol^−1^)	N ^d^ (%)	*v*_D_ ^e^ (m s^−1^)	*P* ^f^ (GPa)	IS ^g^ (J)	FS ^h^ (N)	*Ω*_CO_ ^i^ (%)
**4**	276	1.67	87.95	47.45	6548	18.0	40	360	-
**5**	180	1.854	218.85	41.95	8372	29.5	25	240	0
**6**	192	1.763	116.23	46.66	8076	24.03	25	240	−13.3
**7**	171.2	1.874	397.90	50.25	8939	32.72	1.5	72	−17.7
**8**	204	2.12	−230.81	34.82	7817	25.64	25	240	-
TNT ^j^	295	1.65	−67.0	18.5	6881	19.5	15	360	−24.7
RDX ^k^	204	1.80	70.3	37.8	8795	34.9	7.4	120	0
Pd(N_3_)_2_ ^l^	315	4.80	450.1	28.9	5877	33.4	0.6–4	0.3–0.5	-

^a^ Decomposition temperature (onset) under nitrogen (DSC, 5 °C/min). ^b^ Density measured by gas pycnometer (25 °C). ^c^ Heat of formation. ^d^ Nitrogen content. ^e^ Detonation pressure (calculated with Explo5 v6.05). ^f^ Detonation velocity (calculated with Explo5 6.05). ^g^ Impact sensitivity. ^h^ Friction sensitivity. ^i^ Oxygen balances for C_a_H_b_O_c_N_d_, 1600(c − a − b/2)/M_W_; M_W_ = Molecular weight—based on CO formation. ^j^ ref. [44]. ^k^ ref. [45]. ^l^ ref. [46].

## Data Availability

The raw/processed data required to reproduce these findings will be shared by the corresponding author upon reasonable request.

## Supplementary Materials

The following supporting information can be downloaded at https://www.mdpi.com/article/10.3390/molecules30214224/s1, Figure S1 and Tables S1–S7: crystal structure data; Figures S2–S6: DSC plot; Figures S7–S22: ^1^H and ^13^C NMR spectra. References [37,38,39,40,41,42] are cited in the Supplementary Materials.
