# Supplementary material for: Tri-1,3,4-Oxadiazoles Modified with Nitroimine: Balancing Energy, Sensitivity, and Thermal Stability
Source: Molecules. 2025 Oct 29;30(21):4224. doi: 10.3390/molecules30214224 (PMC12608635; doi:10.3390/molecules30214224)
Supplement: Supplementary file 1 [file molecules-30-04224-s001.zip › molecules-3903416-supplementary.pdf]

Tri-1,3,4-Oxadiazoles Modified with Nitroimine: Balancing
Energy, Sensitivity, and Thermal Stability

Fangming Chen †, Qiong Yu *,†, Lei Li, Kejia Peng, Chenguang Zhu and Wenbin Yi *

School of Chemistry and Chemical Engineering, Nanjing University of Science and Technology, Nanjing 210094, China; chenfm@njust.edu.cn (F.C.); 123103222263@njust.edu.cn (L.L.); 230997@muctr.ru (K.P.); zcg_lnkz@163.com (C.Z.)

* Correspondence: qyu@njust.edu.cn (Q.Y.); yiwb@njust.edu.cn (W.Y.); Tel.: +86-18114015326 (Q.Y.); +86-13951830425 (W.Y.)

† These authors contributed equally to this work.

Table of Contents

1. Crystallographic data.....S1

2. Theoretical calculation.....S6

3. DSC plot of the title compounds.....S11

4. NMR spectra of all new compounds.....S14

X-Ray Crystal structure details

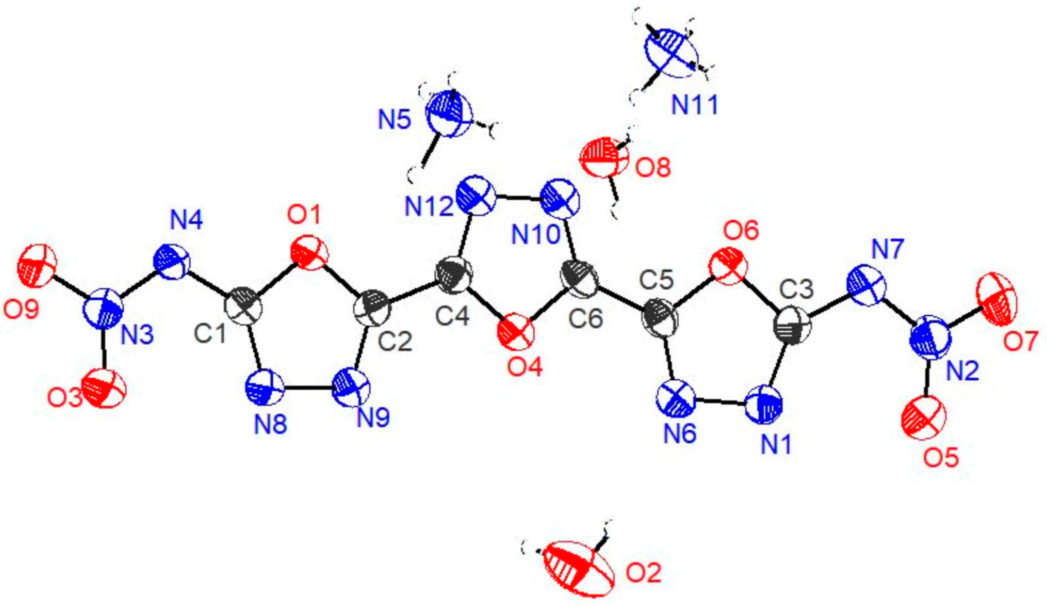

Figure S1: Molecular structure of **6**·$2H_2O$.

Table S1. Crystallographic data for **6**·$2H_2O$.

Identification code	**6**·$2H_2O$
CCDC number	2417139
Empirical formula	$C_6H_{12}N_{12}O_9$
Temperature/K	273.15
Crystal system	monoclinic
Space group	$P2_1/c$
a/Å	6.5250(17)
b/Å	16.600(5)
c/Å	14.397(4)
α/°	90
β/°	97.341(8)
γ/°	90
Volume/Å^3	1546.6(8)
Z	4
ρ_{calc}g/cm^3	1.702
μ/mm^{-1}	0.156
F(000)	816.0
Crystal size/mm^3	0.29×0.18×0.09
Radiation	MoKα(λ=0.71073)
2Θ range for date collected/°	4.908 to 46.462
Index ranges	-7≤h≤7, -18≤k≤8, -15≤l≤15
Reflections collected	23537

Identification code	**6**·$2H_2O$
Independent reflections	2213 [R_{int} = 0.1219, R_{sigma} = 0.0611]
Date/restraints/parameters	2213/0/266
Goodness-of-fit on F^2	1.059
Final R indexes [I>=2σ(I)]	R_1= 0.0499, wR_2= 0.1304
Final R indexes [all date]	R_1= 0.0632, wR_2= 0.1415
Largest diff. peak/hole / e Å^3	0.34/-0.51

Table S2. Fractional □tomic Coordinates ($\times 10^4$) and Equivalent Isotropic Displacement Parameters (Å$^2 \times 10^3$) for **6**·$2H_2O$. U_{eq} is defined as 1/3 of the trace of the orthogonalized U_{IJ} tensor.

Atom	Atom	Length/Å	Atom	Atom	Length/Å
O_1	C_1	1.369(3)	N_3	N_4	1.333(3)
O_1	C_2	1.351(3)	N_4	C_1	1.354(4)
O_3	N_3	1.232(3)	N_6	C_5	1.272(4)
O_4	C_4	1.355(3)	N_7	C_3	1.344(4)
O_4	C_6	1.349(3)	N_8	N_9	1.412(3)
O_5	N_2	1.241(3)	N_8	C_1	1.297(4)
O_6	C_3	1.383(3)	N_9	C_2	1.272(4)
O_6	C_5	1.359(3)	N_{10}	N_{12}	1.402(4)
O_7	N_2	1.237(3)	N_{10}	C_6	1.284(4)
O_9	N_3	1.261(3)	N_{12}	C_4	1.283(4)
N_1	N_6	1.410(3)	C_2	C_4	1.443(4)
N_1	C_3	1.304(4)	C_5	C_6	1.439(4)
N_2	N_7	1.346(4)			

Table S3. □nisotropic Displacement Parameters (Å^2×10^3) for **6**·$2H_2O$. The □nisotropic displacement factor exponent takes the form $-2\pi^2[h^2a^{*2}U_{11}+2hka^*b^*U_{12}+\ldots]$.

Atom	Atom	Atom	Angle/°	Atom	Atom	Atom	Angle/°
C_2	O_1	C_1	101.9(2)	N_8	C_1	O_1	112.4(2)
C_6	O_4	C_4	101.0(2)	N_8	C_1	N_4	136.5(3)
C_5	O_6	C_3	102.1(2)	O_1	C_2	C_4	118.2(2)
C_3	N_1	N_6	106.3(2)	N_9	C_2	O_1	114.1(3)
O_5	N_2	N_7	122.9(3)	N_9	C_2	C_4	127.7(3)
O_7	N_2	O_5	122.4(3)	N_1	C_3	O_6	111.4(2)
O_7	N_2	N_7	114.7(3)	N_1	C_3	N_7	137.2(3)
O_3	N_3	O_9	121.4(2)	N_7	C_3	O_6	111.3(2)
O_3	N_3	N_4	123.8(3)	O_4	C_4	C_2	117.1(2)
O_9	N_3	N_4	114.8(2)	N_{12}	C_4	O_4	114.1(3)
N_3	N_4	C_1	115.9(2)	N_{12}	C_4	C_2	128.8(3)
C_5	N_6	N_1	106.3(2)	O_6	C_5	C_6	118.7(3)
C_3	N_7	N_2	115.9(2)	N_6	C_5	O_6	113.9(3)

Atom	Atom	Atom	Angle/°	Atom	Atom	Atom	Angle/°
C_1	N_8	N_9	105.6(2)	N_6	C_5	C_6	127.4(3)
C_2	N_9	N_8	106.0(2)	O_4	C_6	C_5	117.3(3)
C_6	N_{10}	N_{12}	105.6(2)	N_{10}	C_6	O_4	114.0(3)
C_4	N_{12}	N_{10}	105.3(2)	N_{10}	C_6	C_5	128.6(3)
N_4	C_1	O_1	111.1(2)				

Table S4. Bond Lengths for **6**·$2H_2O$**.**

A	B	C	D	Angle/°	A	B	C	D	Angle/°
O_1	C_2	C_4	O_4	179.1(2)	N_9	C_2	C_4	O_4	-1.3(4)
O_1	C_2	C_4	N_{12}	-0.5(4)	N_9	C_2	C_4	N_{12}	179.1(3)
O_3	N_3	N_4	C_1	1.8(4)	N_{10}	N_{12}	C_4	O_4	0.2(3)
O_5	N_2	N_7	C_3	-3.2(4)	N_{10}	N_{12}	C_4	C_2	179.8(3)
O_6	C_5	C_6	O_4	-174.9(2)	N_{12}	N_{10}	C_6	O_4	0.3(3)
O_6	C_5	C_6	N_{10}	4.1(4)	N_{12}	N_{10}	C_6	C_5	-178.7(3)
O_7	N_2	N_7	C_3	176.8(3)	C_1	O_1	C_2	N_9	-0.6(3)
O_9	N_3	N_4	C_1	-178.5(2)	C_1	O_1	C_2	C_4	179.1(2)
N_1	N_6	C_5	O_6	-0.3(3)	C_1	N_8	N_9	C_2	0.4(3)
N_1	N_6	C_5	C_6	-179.2(3)	C_2	O_1	C_1	N_4	-178.5(2)
N_2	N_7	C_3	O_6	-174.8(2)	C_2	O_1	C_1	N_8	0.8(3)
N_2	N_7	C_3	N_1	4.7(5)	C_3	O_6	C_5	N_6	0.4(3)
N_3	N_4	C_1	O_1	179.7(2)	C_3	O_6	C_5	C_6	179.3(2)
N_3	N_4	C_1	N_8	0.7(5)	C_3	N_1	N_6	C_5	0.1(3)
N_6	N_1	C_3	O_6	0.1(3)	C_4	O_4	C_6	N_{10}	-0.2(3)
N_6	N_1	C_3	N_7	-179.4(3)	C_4	O_4	C_6	C_5	179.0(2)
N_6	C_5	C_6	O_4	3.9(4)	C_5	O_6	C_3	N_1	-0.2(3)
N_6	C_5	C_6	N_{10}	-177.1(3)	C_5	O_6	C_3	N_7	179.4(2)
N_8	N_9	C_2	O_1	0.1(3)	C_6	O_4	C_4	N_{12}	0.0(3)
N_8	N_9	C_2	C_4	-179.5(3)	C_6	O_4	C_4	C_2	-179.7(2)
N_9	N_8	C_1	O_1	-0.8(3)	C_6	N_{10}	N_{12}	C_4	-0.3(3)
N_9	N_8	C_1	N_4	178.3(3)					

Table S5. Bond □ngles for **6**·$2H_2O$.

Atom	Atom	Atom	Angle/°	Atom	Atom	Atom	Angle/°
C_2	O_1	C_1	101.9(2)	N_8	C_1	O_1	112.4(2)
C_6	O_4	C_4	101.0(2)	N_8	C_1	N_4	136.5(3)
C_5	O_6	C_3	102.1(2)	O_1	C_2	C_4	118.2(2)
C_3	N_1	N_6	106.3(2)	N_9	C_2	O_1	114.1(3)
O_5	N_2	N_7	122.9(3)	N_9	C_2	C_4	127.7(3)
O_7	N_2	O_5	122.4(3)	N_1	C_3	O_6	111.4(2)
O_7	N_2	N_7	114.7(3)	N_1	C_3	N_7	137.2(3)

O_3	N_3	O_9	121.4(2)	N_7	C_3	O_6	111.3(2)
O_3	N_3	N_4	123.8(3)	O_4	C_4	C_2	117.1(2)
O_9	N_3	N_4	114.8(2)	N_{12}	C_4	O_4	114.1(3)
N_3	N_4	C_1	115.9(2)	N_{12}	C_4	C_2	128.8(3)
C_5	N_6	N_1	106.3(2)	O_6	C_5	C_6	118.7(3)
C_3	N_7	N_2	115.9(2)	N_6	C_5	O_6	113.9(3)
C_1	N_8	N_9	105.6(2)	N_6	C_5	C_6	127.4(3)
C_2	N_9	N_8	106.0(2)	O_4	C_6	C_5	117.3(3)
C_6	N_{10}	N_{12}	105.6(2)	N_{10}	C_6	O_4	114.0(3)
C_4	N_{12}	N_{10}	105.3(2)	N_{10}	C_6	C_5	128.6(3)
N_4	C_1	O_1	111.1(2)				

Table S6. Torsion □ngles for **6**·$2H_2O$**.**

A	B	C	D	Angle/°	A	B	C	D	Angle/°
O_1	C_2	C_4	O_4	179.1(2)	N_9	C_2	C_4	O_4	-1.3(4)
O_1	C_2	C_4	N_{12}	-0.5(4)	N_9	C_2	C_4	N_{12}	179.1(3)
O_3	N_3	N_4	C_1	1.8(4)	N_{10}	N_{12}	C_4	O_4	0.2(3)
O_5	N_2	N_7	C_3	-3.2(4)	N_{10}	N_{12}	C_4	C_2	179.8(3)
O_6	C_5	C_6	O_4	-174.9(2)	N_{12}	N_{10}	C_6	O_4	0.3(3)
O_6	C_5	C_6	N_{10}	4.1(4)	N_{12}	N_{10}	C_6	C_5	-178.7(3)
O_7	N_2	N_7	C_3	176.8(3)	C_1	O_1	C_2	N_9	-0.6(3)
O_9	N_3	N_4	C_1	-178.5(2)	C_1	O_1	C_2	C_4	179.1(2)
N_1	N_6	C_5	O_6	-0.3(3)	C_1	N_8	N_9	C_2	0.4(3)
N_1	N_6	C_5	C_6	-179.2(3)	C_2	O_1	C_1	N_4	-178.5(2)
N_2	N_7	C_3	O_6	-174.8(2)	C_2	O_1	C_1	N_8	0.8(3)
N_2	N_7	C_3	N_1	4.7(5)	C_3	O_6	C_5	N_6	0.4(3)
N_3	N_4	C_1	O_1	179.7(2)	C_3	O_6	C_5	C_6	179.3(2)
N_3	N_4	C_1	N_8	0.7(5)	C_3	N_1	N_6	C_5	0.1(3)
N_6	N_1	C_3	O_6	0.1(3)	C_4	O_4	C_6	N_{10}	-0.2(3)
N_6	N_1	C_3	N_7	-179.4(3)	C_4	O_4	C_6	C_5	179.0(2)
N_6	C_5	C_6	O_4	3.9(4)	C_5	O_6	C_3	N_1	-0.2(3)
N_6	C_5	C_6	N_{10}	-177.1(3)	C_5	O_6	C_3	N_7	179.4(2)
N_8	N_9	C_2	O_1	0.1(3)	C_6	O_4	C_4	N_{12}	0.0(3)
N_8	N_9	C_2	C_4	-179.5(3)	C_6	O_4	C_4	C_2	-179.7(2)
N_9	N_8	C_1	O_1	-0.8(3)	C_6	N_{10}	N_{12}	C_4	-0.3(3)
N_9	N_8	C_1	N_4	178.3(3)					

Table S7. Hydrogen □tom Coordinates (Å×10^4) and Isotropic Displacement Parameters (Å^2×10^3) for **6**·$2H_2O$**.**

Atom	*x*	*y*	*z*	U(eq)
H_2□	2318.05	3659.57	2718.03	146

**Atom**	***x***	***y***	***z***	**U(eq)**
H_{2B}	2174.72	4388.06	3112.39	146
$H_{8□}$	8980.12	3963.97	6495.96	78
H_{8B}	10092.02	3981.32	7348.28	78
$H_{11□}$	6719.53	4516.46	8139.94	70
H_{11B}	5012.63	4207.26	8591.74	70
H_{11C}	6883.33	3731.96	8591.74	70
H_{11D}	5497.93	3843.96	7727.64	70
$H_{5□}$	580(80)	1860(30)	6410(40)	125(19)
H_{5B}	-30(70)	2510(30)	7060(30)	75(14)
H_{5C}	1440(80)	2060(30)	7510(40)	99(19)
H_{5D}	-770(80)	1770(30)	7330(30)	82(16)

Theoretical calculations

The calculation was carried out using the Gaussian 16 program suite.[1] The geometric optimization and frequency analyses of compounds were fulfilled using the B3-LYP[2] functional with 6-31G**[3,4] basis set, and single energy points were calculated at the M06-2X/DEF2-TZVP level. □ll the optimized structures were verified to be true local energy minima on the potential energy surface without imaginary frequencies. The predictions of heats of formation ($\Delta_f H$) used designed isodesmic reactions. The isodesmic reaction processes in which the number of each kind of formal bond is conserved were used, along with the application of the bond separation reaction (BSR) rules. The molecule was broken down into a set of two heavy-atom molecules containing the same component bonds. The isodesmic reactions used to derive the $\Delta_f H_{gas}$ of compounds **4**, **5** and the anion of **5** are shown in Scheme Sl. The total energies (E0), zero-point correction (ZPE), thermal corrections (H_T), and the experimental/calculated HOF values of the reference compounds used in isodesmic reactions are given in Tables S8 and S9.

□t 298K, the isodesmic reactions for calculating the enthalpy of formation ($\Delta_f H_{298K}$) of target compounds generally follow the two forms below:

X-nR+$n$$CH_4$ → X +$n$$CH_3$R S1

X-nR+$n$$NH_3$ → X +$n$$NH_2$R S2

where X represents the parent skeleton (delocalized bond system or cage-like structure); R denotes the substituent; and CH_4, NH_3, CH_3R, and NH_2R are reference substances. For isodesmic reactions (S1) and (S2), the reaction enthalpy ($\Delta_r H_{298K}$) at 298K can be expressed by the following equation:

$$\Delta_r H_{298K} = \sum \Delta_f H_{298K}(P) - \sum \Delta_f H_{298K}(R) \quad \text{S3}$$

In Equation (S3), $\Delta_f H_{298K}(R)$ and $\Delta_f H_{298K}(P)$ represent the enthalpy of formation of reactants and products at 298K, respectively.

When $\Delta_r H_{298K}$ is known, the enthalpy of formation of the target compound can be calculated using Equation (S3). Meanwhile, $\Delta_r H_{298K}$ itself can be computed via the formula below:

$$\Delta_r H_{298K} = \Delta E_{298K} + \Delta(PV) = \Delta E_0 + \Delta ZPE + \Delta H_T + \Delta nRT \quad \text{S4}$$

In Equation (S4), ΔE_0 refers to the difference in total energy between products and reactants at 0 K; ΔZPE denotes the difference in zero-point energy (ZPE) between products and reactants at 0 K; and ΔH_T is the temperature correction term from 0 K to 298 K. In an ideal gas reaction, $\Delta(PV) = \Delta nRT$ (where Δn is the change in the amount of gaseous substances, R is the gas constant, and T is the temperature. For isodesmic reactions (S1) and (S2), Δn=0, so $\Delta(PV)$=0.

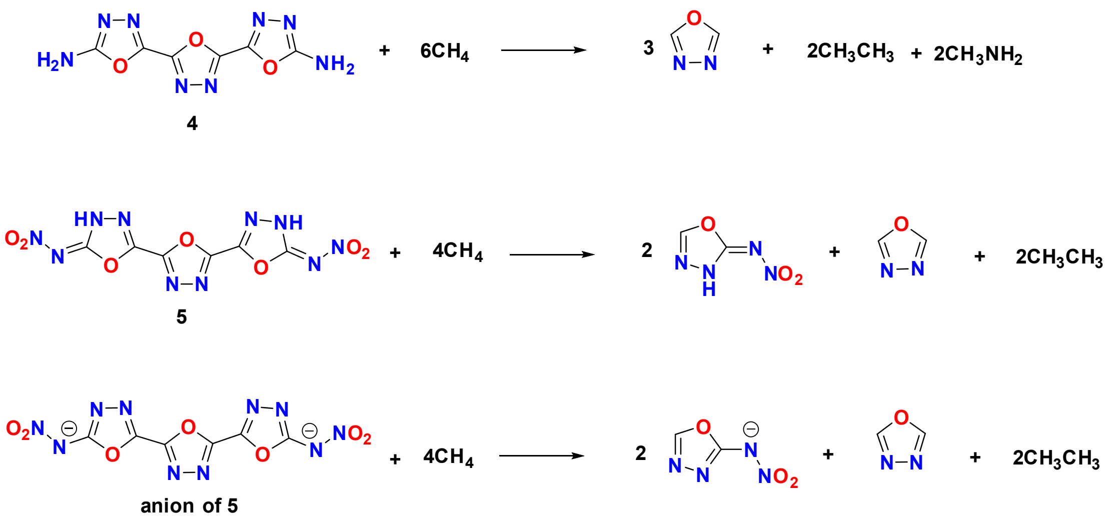

Scheme S1. Isodesmic reactions for the heats of formation.

Table S8. Calculated zero-point energy (ZPE), thermal correction to enthalpy (H_T), total energy (E_0) and gas-phase heats of formation (HOF (gas))

Compound	ZPE (Hartree/Particle)	H_T (Hartree/Particle)	E_0 (Hartree/Particle)	$\Delta_f H$ (gas) (kJ·mol^{-1})
CH_4	0.045926	0.00381	-40.45216502	-74.9
CH_3NH_2	0.064218	0.004334	-95.77492423	-23.5
CH_3CH_3	0.074924	0.00442	-79.72616407	-84.0
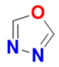	0.046473	0.004382	-262.0503897	71.68
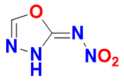	0.06637	0.007649	-521.9531	123.46
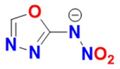	0.052498	0.007647	-521.4263	-55.16
anion	0.111504	0.018537	-1302.5548	97.17
**4**	0.133024	0.014516	-894.5166759	248.23
**5**	0.138723	0.018629	-1303.624936	409.74

The sublimation enthalpies of compounds **4** and **5** were calculated using the Geometrical Fragment (GF) approach[5,6], with values of 160.28 kJ·mol^{-1} and 190.89 kJ·mol^{-1}, respectively. The solid-state heats of formation of compounds **4** and **5** were computed using the following formula: $\Delta H_f = \Delta H_f(g) - \Delta H_{sub}$.

Table S9. Energy content of salts **6-8.**

Compound	$\Delta_f H_a$[a]	$\Delta_f H_c$[b]	H_L[c]	$\Delta_f H_{salt}$[d]

	97.17	634.87	1250.69	116.23
	97.17	771.21	1241.70	397.90
	97.17	504.33	1336.63	-230.81

[a] Heat of formation of anion (kJ mol^{-1}). [b] Heat of formation of cation (kJ mol^{-1}). [c] Lattice energy (kJ mol^{-1}). [d] Heat of formation of salt (kJ mol^{-1}).

The geometries and XYZ coordinates of the neutral molecule and anion are as follows:

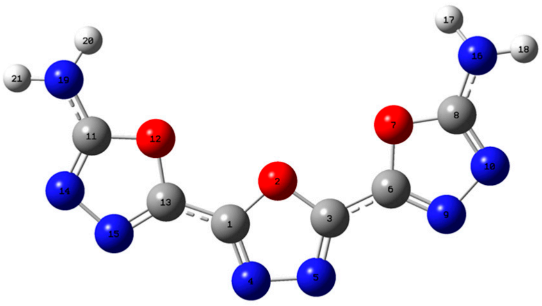

Figure S2. The optimized configuration of **4**.

Table S10. The optimized Cartesian coordinates of compound **4**.

Center Number	□tomic Number	Forces (Hartrees/Bohr) X	Y	Z
1	6	0.000006655	-0.000025616	-0.000000644
2	8	-0.000014447	0.000019243	-0.000002644
3	6	0.000021174	-0.000015244	-0.000004491
4	7	0.000021908	0.000030738	-0.000006999
5	7	-0.000035947	-0.000010591	0.000008331
6	6	0.000004280	-0.000010109	0.000006968
7	8	-0.000018641	-0.000001678	0.000002763
8	6	-0.000005468	0.000022216	-0.000011591
9	7	0.000010426	-0.000003616	-0.000000984
10	7	0.000007246	-0.000003961	0.000001645
11	6	-0.000022518	0.000010036	-0.000007074

12	8	0.000007391	0.000016918	-0.000004135
13	6	0.000010338	-0.000005781	0.000005363
14	7	0.000002210	-0.000007559	0.000002980
15	7	0.000000172	-0.000010941	0.000001733
16	7	0.000001570	-0.000002876	0.000003937
17	1	0.000004586	0.000002972	-0.000002116
18	1	0.000000022	0.000000425	0.000001622
19	7	0.000003504	-0.000001495	0.000003425
20	1	-0.000004613	-0.000003600	0.000000322
21	1	0.000000154	0.000000519	0.000001587

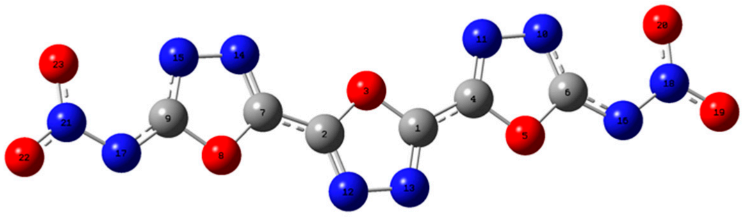

Figure S3. The optimized configuration of the anion of **5**.

Table S11. The optimized Cartesian coordinates of the anion of **5**.

Center Number	□tomic Number	X	Forces (Hartrees/Bohr) Y	Z
1	6	-0.000003446	0.000005366	0.000015999
2	6	-0.000006157	-0.000008022	0.000013942
3	8	0.000003031	-0.000007180	0.000042733
4	6	0.000002301	0.000025049	-0.000002876
5	8	-0.000013125	-0.000069612	0.000004708
6	6	0.000021286	0.000058300	-0.000006946
7	6	-0.000007240	-0.000022053	-0.000010113
8	8	0.000014101	0.000064795	0.000025359
9	6	-0.000002134	-0.000057315	-0.000024711
10	7	0.000000553	0.000024574	0.000024504
11	7	0.000003262	0.000010132	-0.000006613
12	7	-0.000006930	-0.000036742	-0.000008604
13	7	0.000008089	0.000037401	0.000002788
14	7	-0.000000459	-0.000008234	-0.000009435
15	7	-0.000010274	-0.000028874	0.000016292
16	7	-0.000004957	-0.000071863	-0.000046681
17	7	0.000025501	0.000078497	-0.000023578
18	7	-0.000033613	0.000066531	-0.000013231
19	8	0.000019957	-0.000020997	-0.000030938

20	8	0.000008588	-0.000009905	0.000045815
21	7	-0.000055566	-0.000041842	-0.000029882
22	8	0.000028218	0.000019783	-0.000024672
23	8	0.000009016	-0.000007790	0.000046140

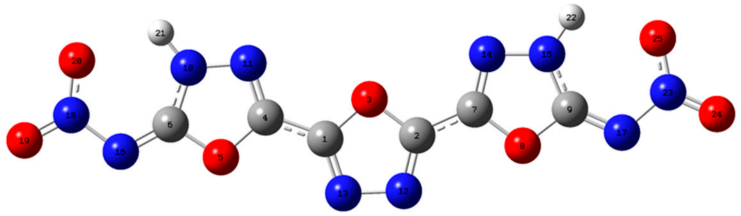

Figure S4. The optimized configuration of **5**.

Table S12. The optimized Cartesian coordinates of compound **5**.

Center Number	□tomic Number	X	Forces (Hartrees/Bohr) Y	Z
1	6	-0.000027473	0.000001004	-0.000011378
2	6	-0.000025072	0.000012857	-0.000009557
3	8	0.000031528	-0.000005503	-0.000005749
4	6	0.000056267	-0.000006376	0.000002878
5	8	-0.000015289	0.000006912	-0.000015672
6	6	-0.000076242	0.000026643	0.000060352
7	6	0.000054188	-0.000016641	0.000001301
8	8	-0.000015825	0.000004269	-0.000016078
9	6	-0.000084032	-0.000011815	0.000054443
10	7	0.000044249	-0.000074843	-0.000039533
11	7	-0.000039267	0.000025237	0.000003316
12	7	0.000010925	0.000006023	0.000016177
13	7	0.000006845	-0.000014118	0.000013083
14	7	-0.000046038	-0.000008187	-0.000001820
15	7	0.000071630	0.000060327	-0.000018764
16	7	0.000070713	0.000020927	-0.000060721
17	7	0.000060849	-0.000027768	-0.000068203
18	7	-0.000111233	-0.000025348	0.000092453
19	8	0.000039160	0.000019730	-0.000008306
20	8	0.000042245	-0.000005044	-0.000046668
21	1	-0.000005214	0.000030622	0.000013470
22	1	-0.000017252	-0.000028807	0.000004338
23	7	-0.000098419	0.000037912	0.000102173
24	8	0.000029119	-0.000029839	-0.000015922
25	8	0.000043637	0.000001826	-0.000045613

DSC plot of the title compounds.

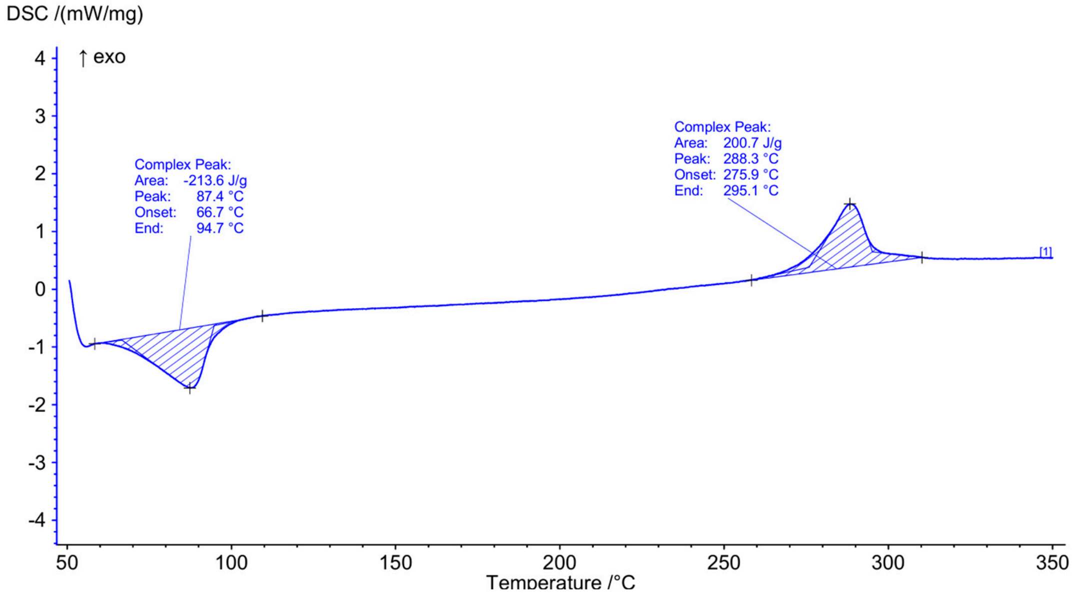

Figure S5. DSC plot of compound **4**.

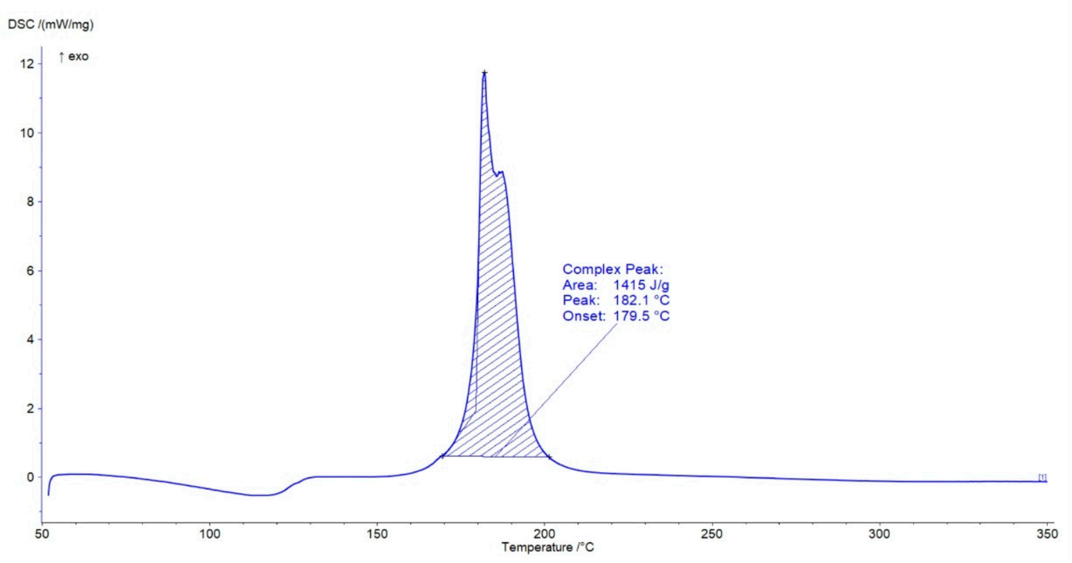

Figure S6. DSC plot of compound **5**.

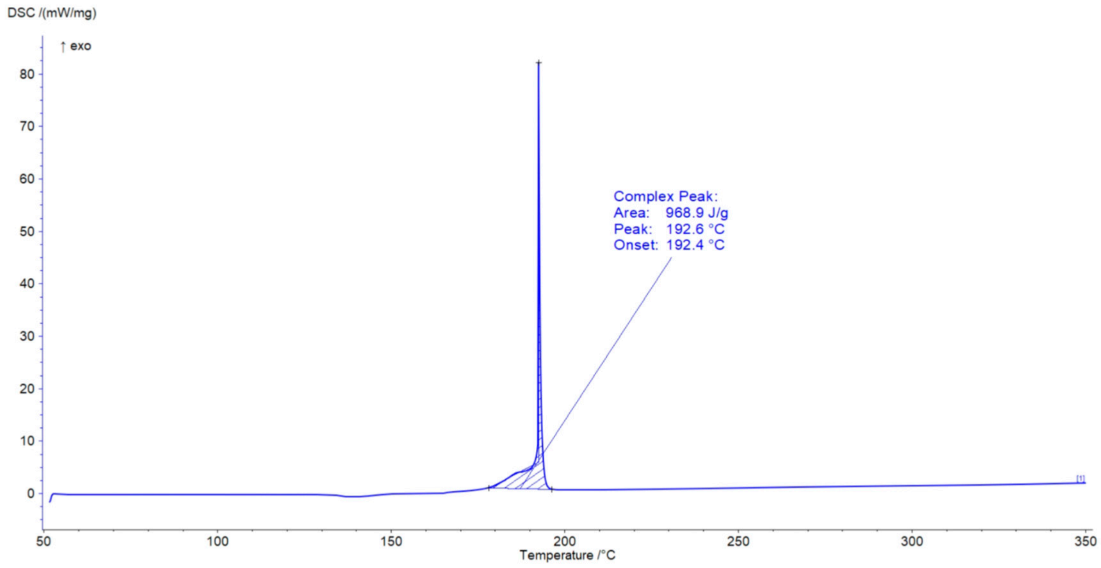

Figure S7. DSC plot of compound **6**.

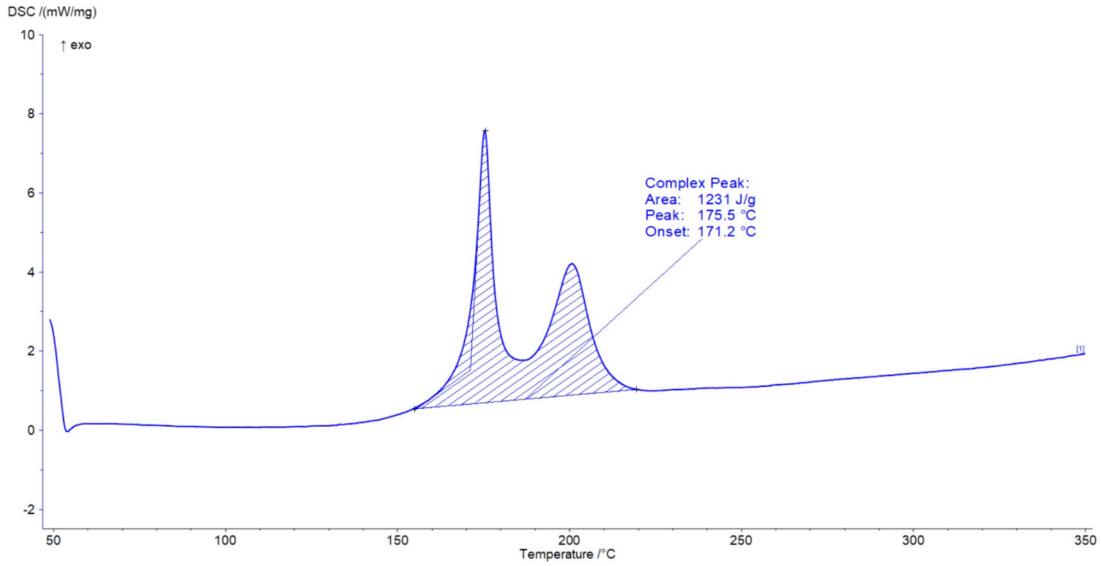

Figure S8. DSC plot of compound **7**.

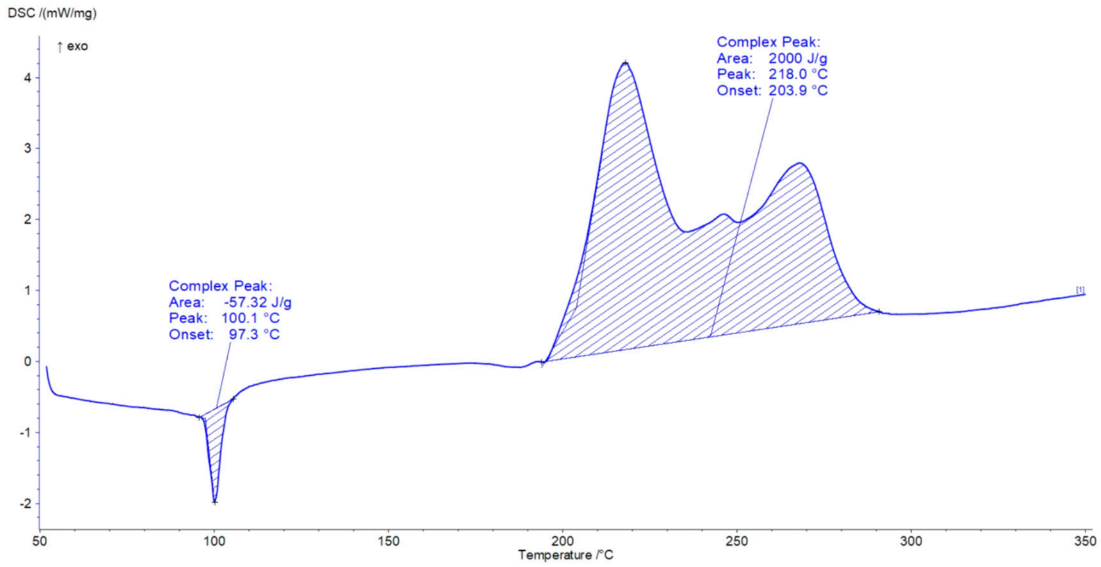

Figure S9. DSC plot of compound **8**.

^{1}H and ^{13}C NMR spectra of all new compounds.

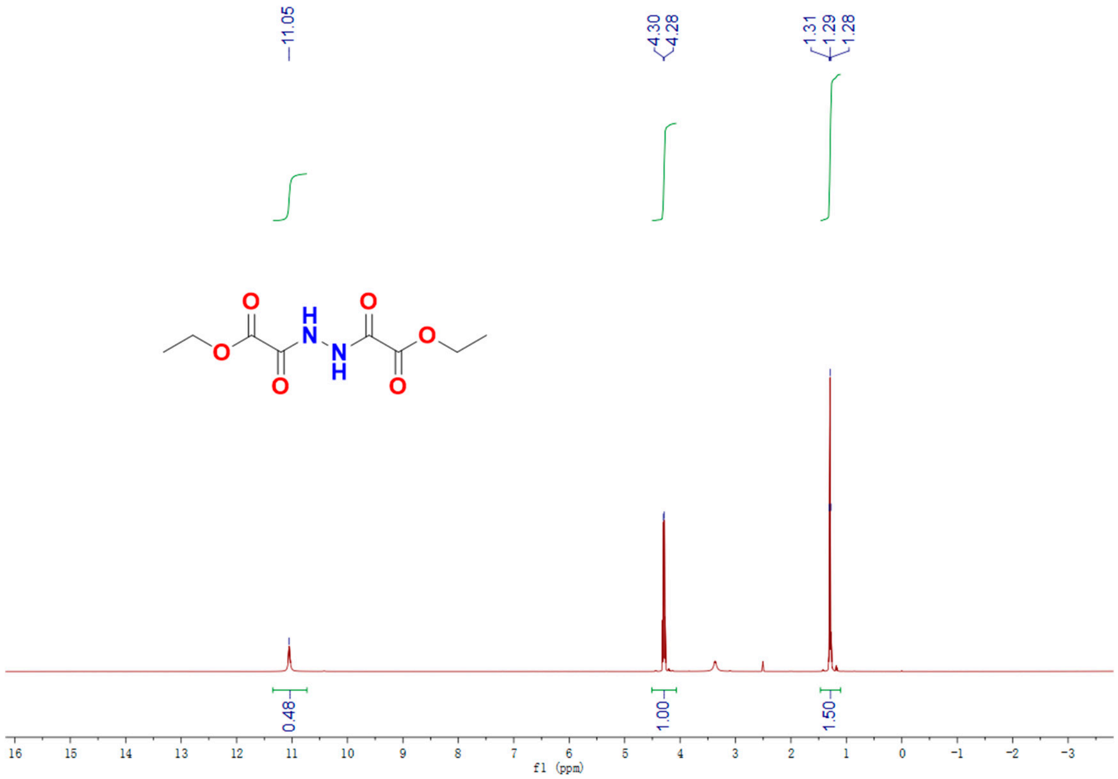

Figure S10. ^{1}H-NMR spectrum of compound **1** in DMSO-d_6.

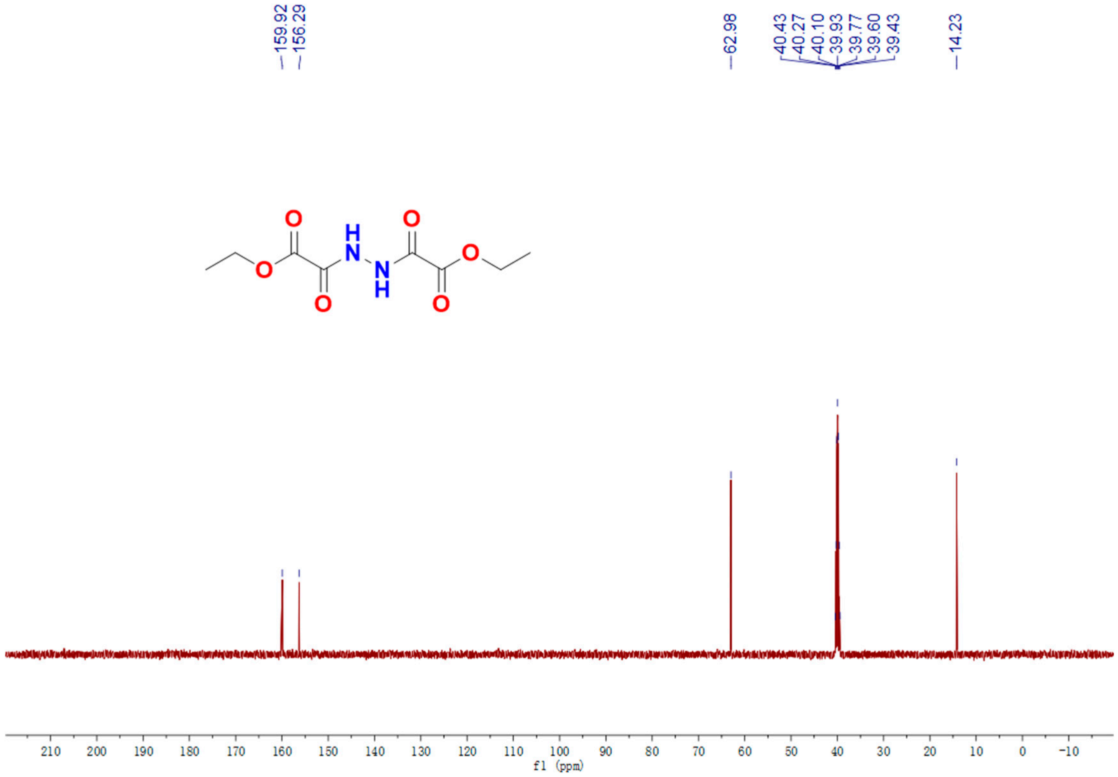

Figure S11. ^{13}C-NMR spectrum of compound **1** in DMSO-d_6.

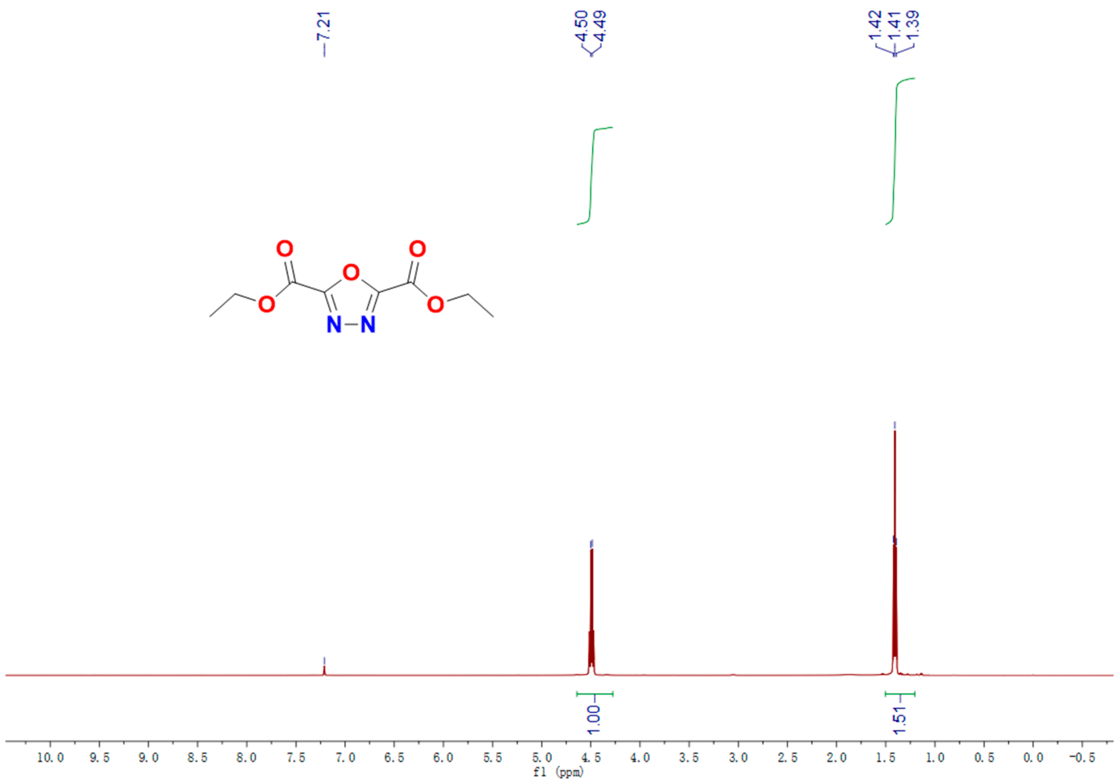

Figure S12. ^{1}H-NMR spectrum of compound **2** in $CDCl_3$.

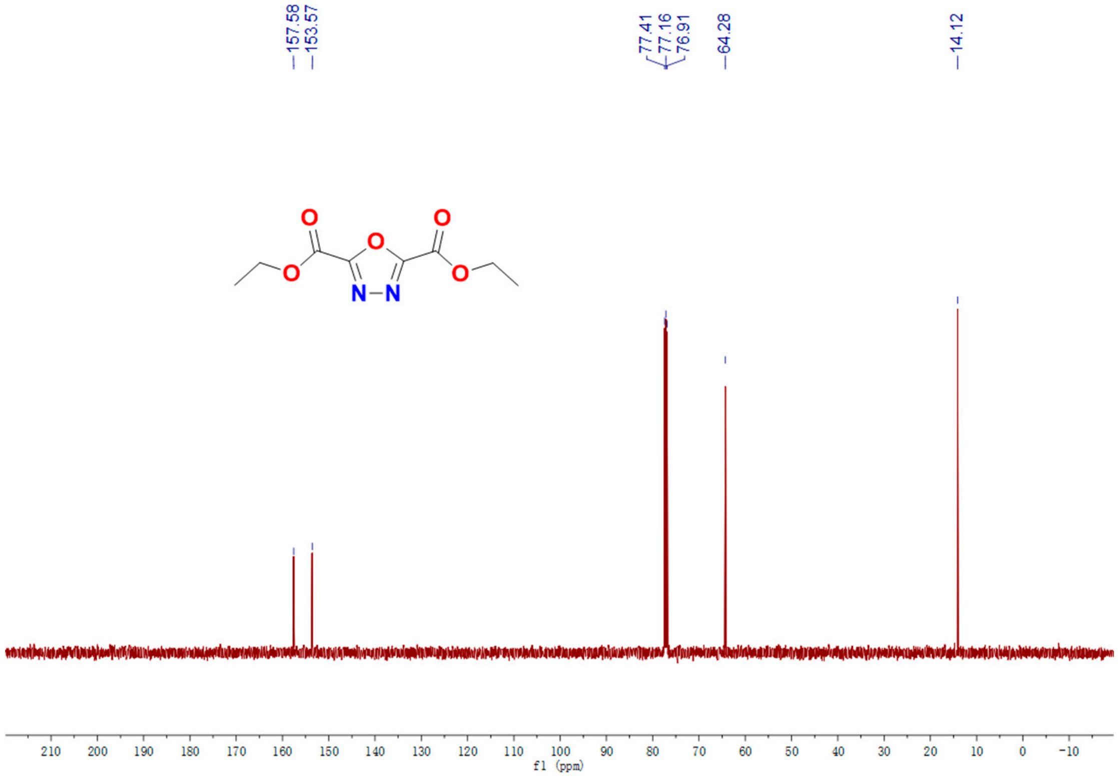

Figure S13. ^{13}C-NMR spectrum of compound **2** in $CDCl_3$.

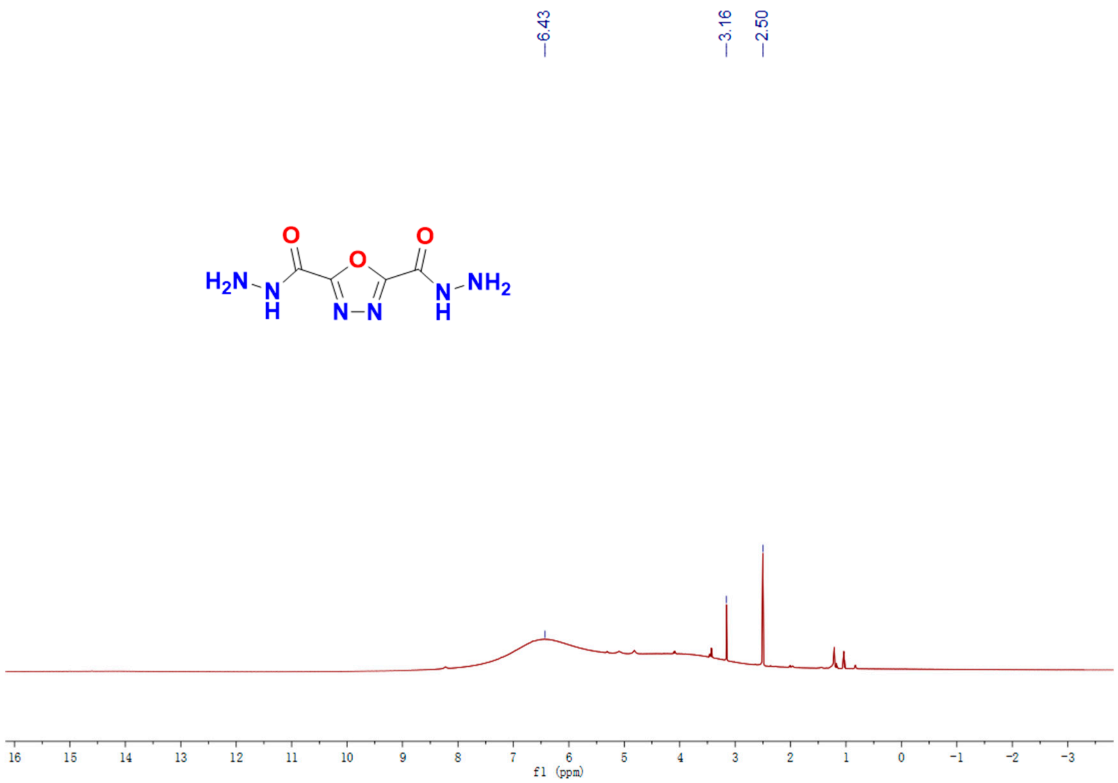

Figure S14. ^{1}H-NMR spectrum of compound **3** in DMSO-d_6.

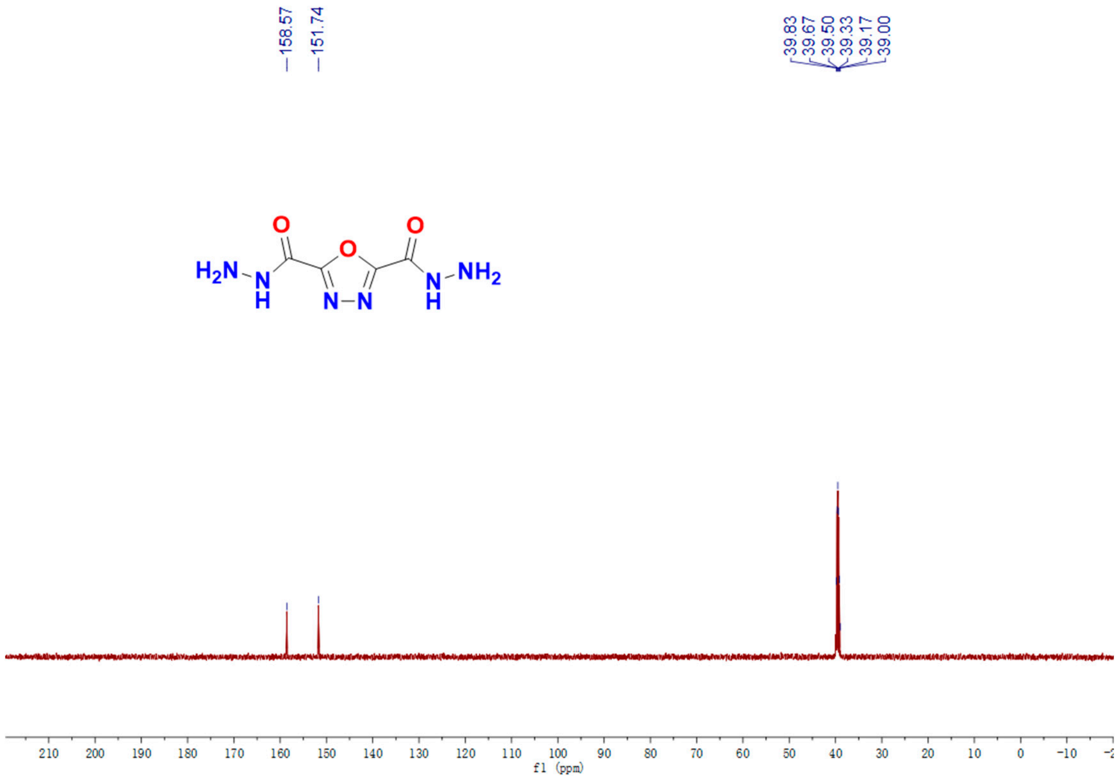

Figure S15. ^{13}C-NMR spectrum of compound **3** in DMSO-d_6.

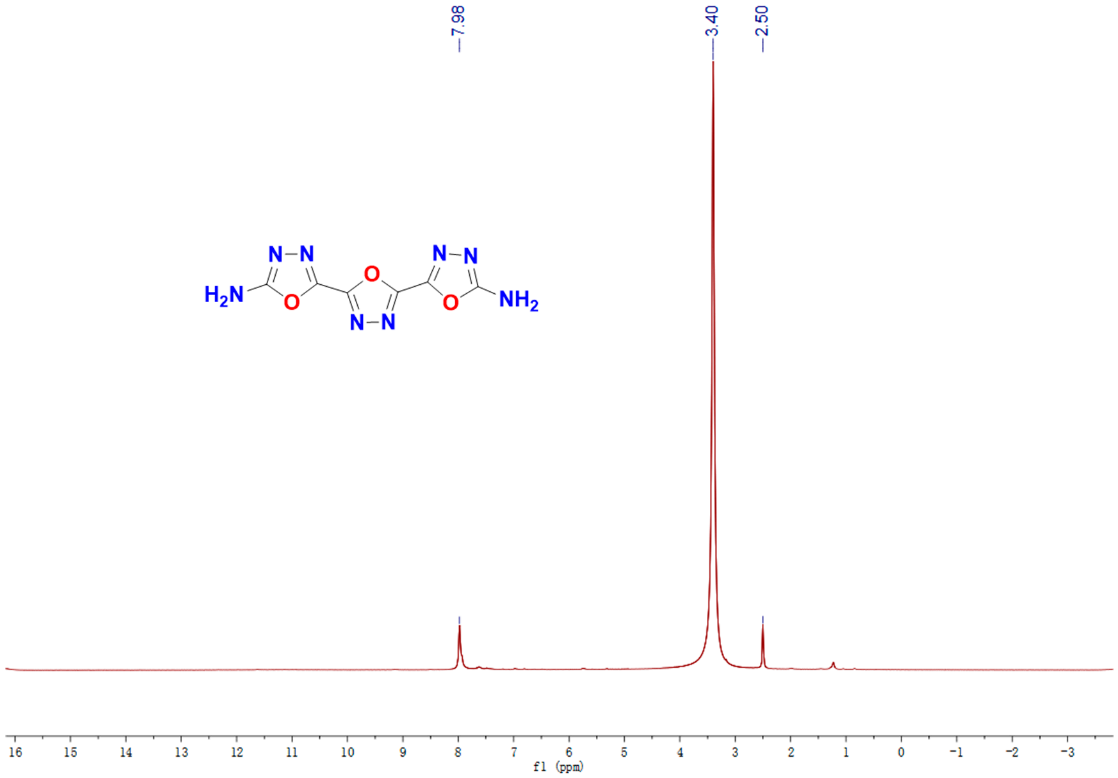

Figure S16. ^{1}H-NMR spectrum of compound **4** in DMSO-d_6.

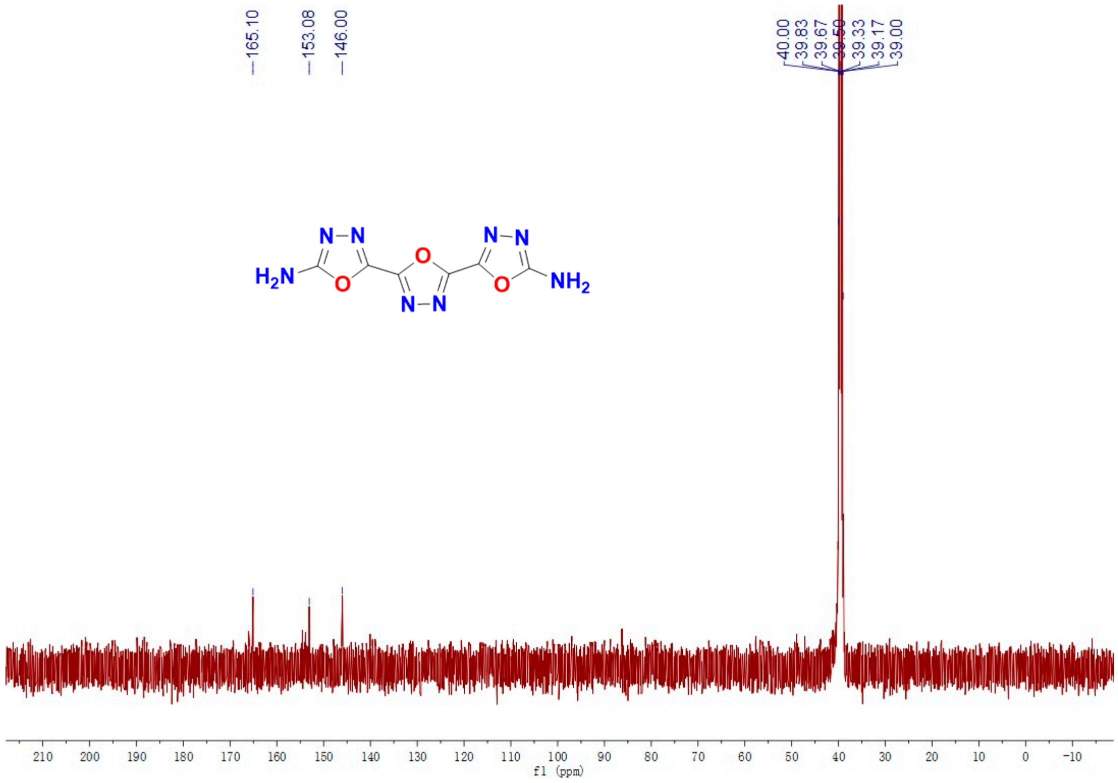

Figure S17. ^{13}C-NMR spectrum of compound **4** in DMSO-d_6.

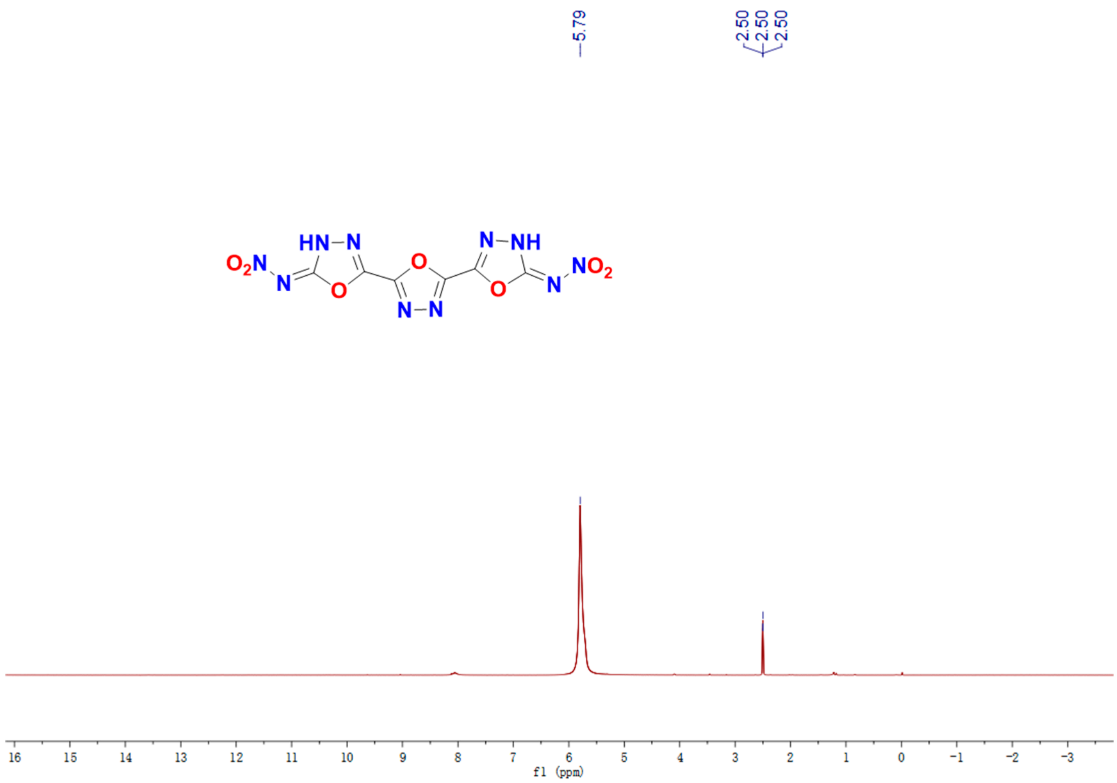

Figure S18. ^{1}H-NMR spectrum of compound **5** in DMSO-d_6.

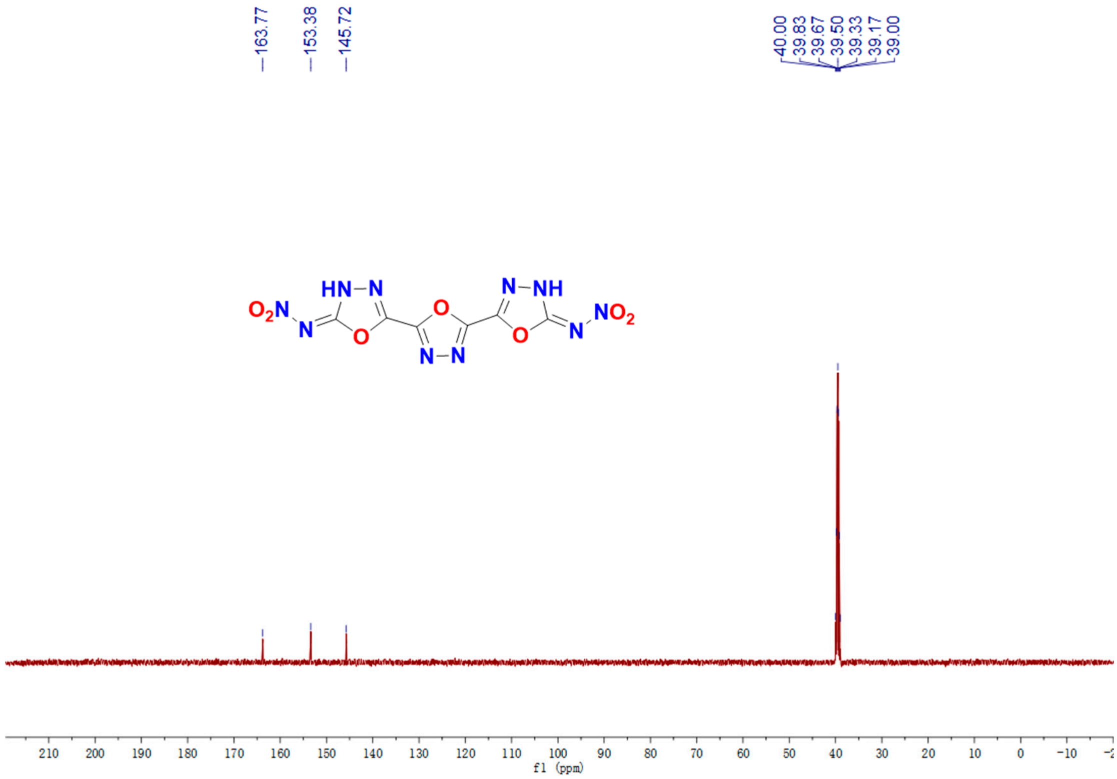

Figure S19. ^{13}C-NMR spectrum of compound **5** in DMSO-d_6.

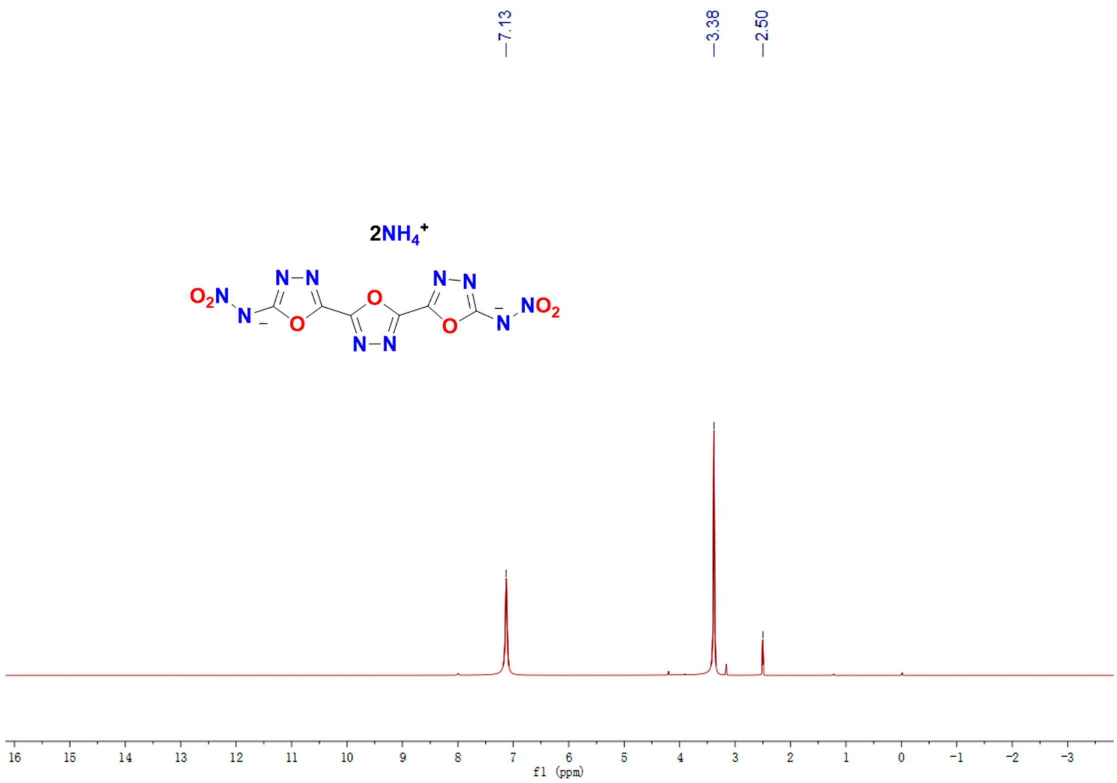

Figure S20. ^{1}H-NMR spectrum of compound **6** in DMSO-*d₆*.

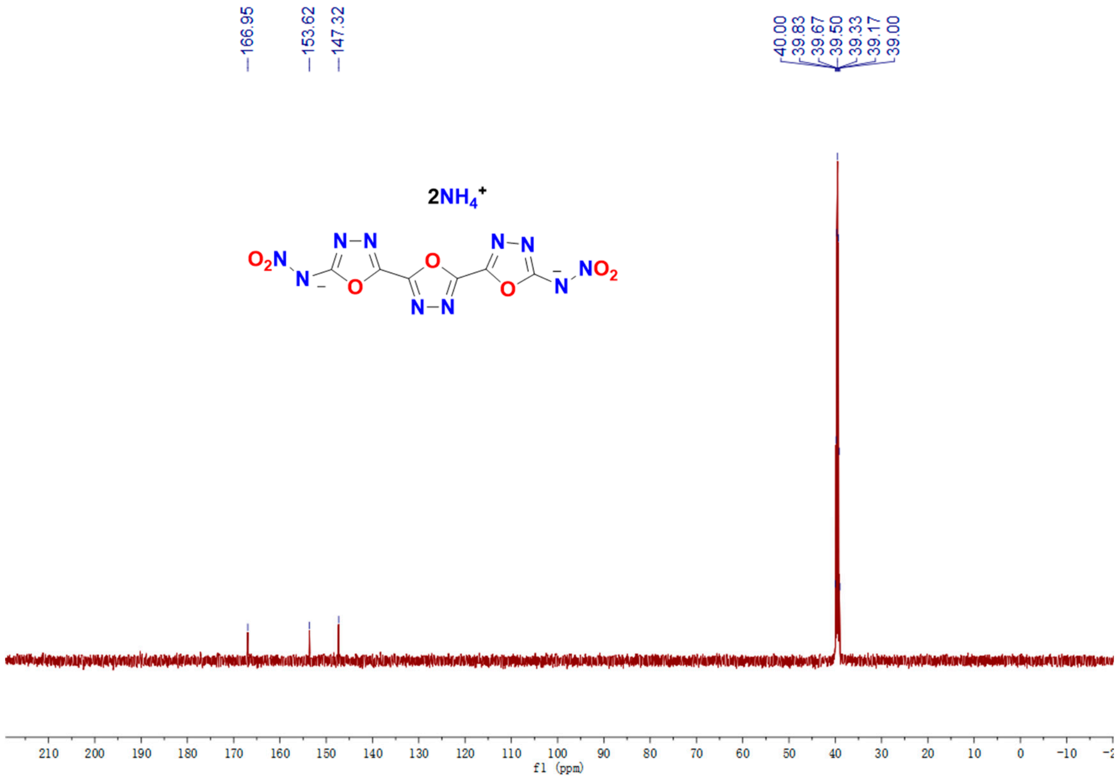

Figure S21. ^{13}C-NMR spectrum of compound **6** in DMSO-*d₆*.

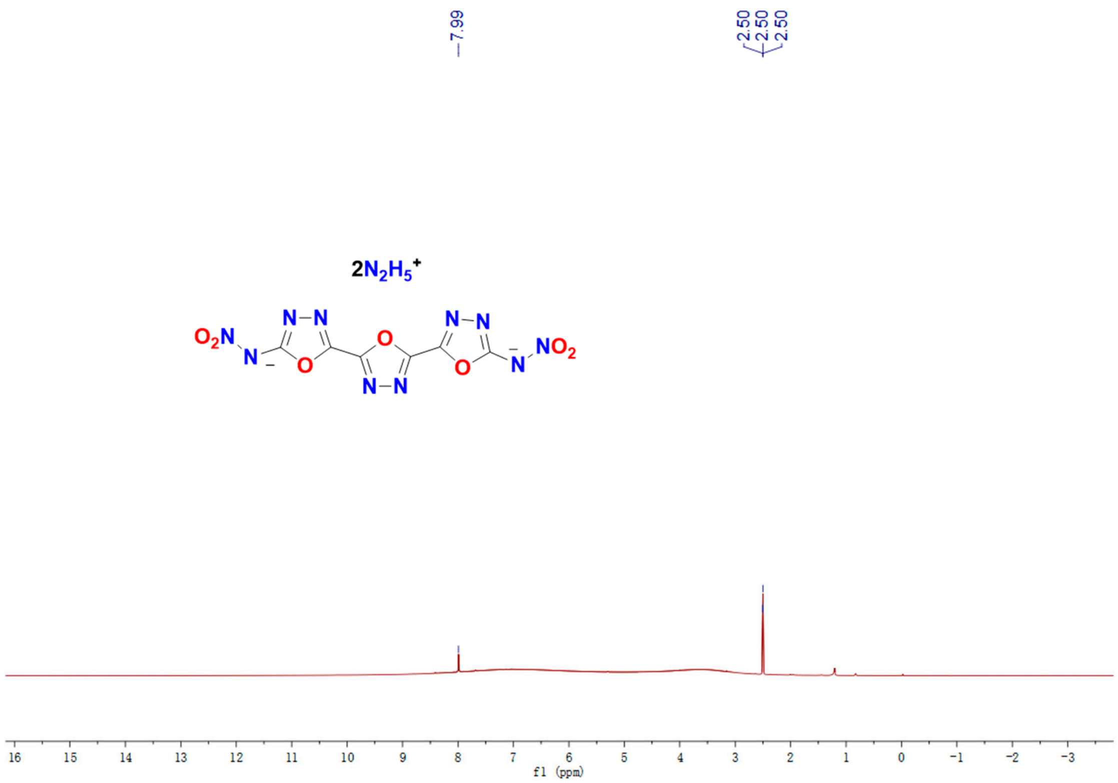

Figure S22. ^{1}H-NMR spectrum of compound **7** in DMSO-d_6.

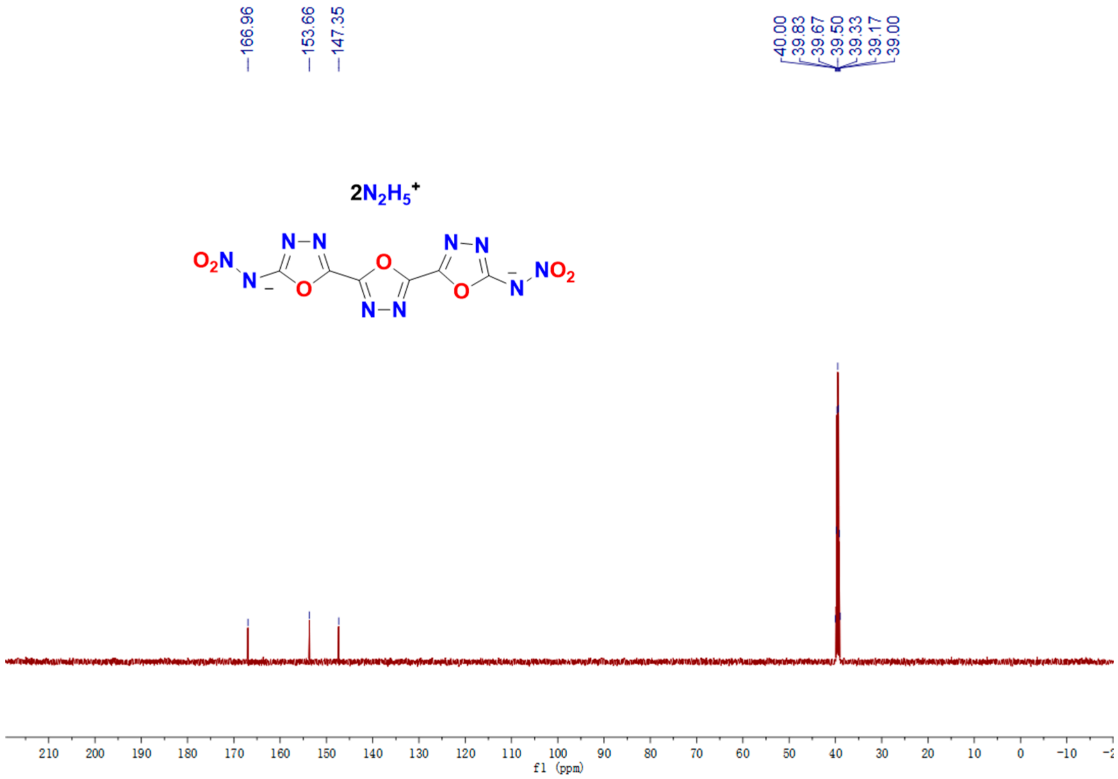

Figure S23. ^{13}C-NMR spectrum of compound **7** in DMSO-d_6.

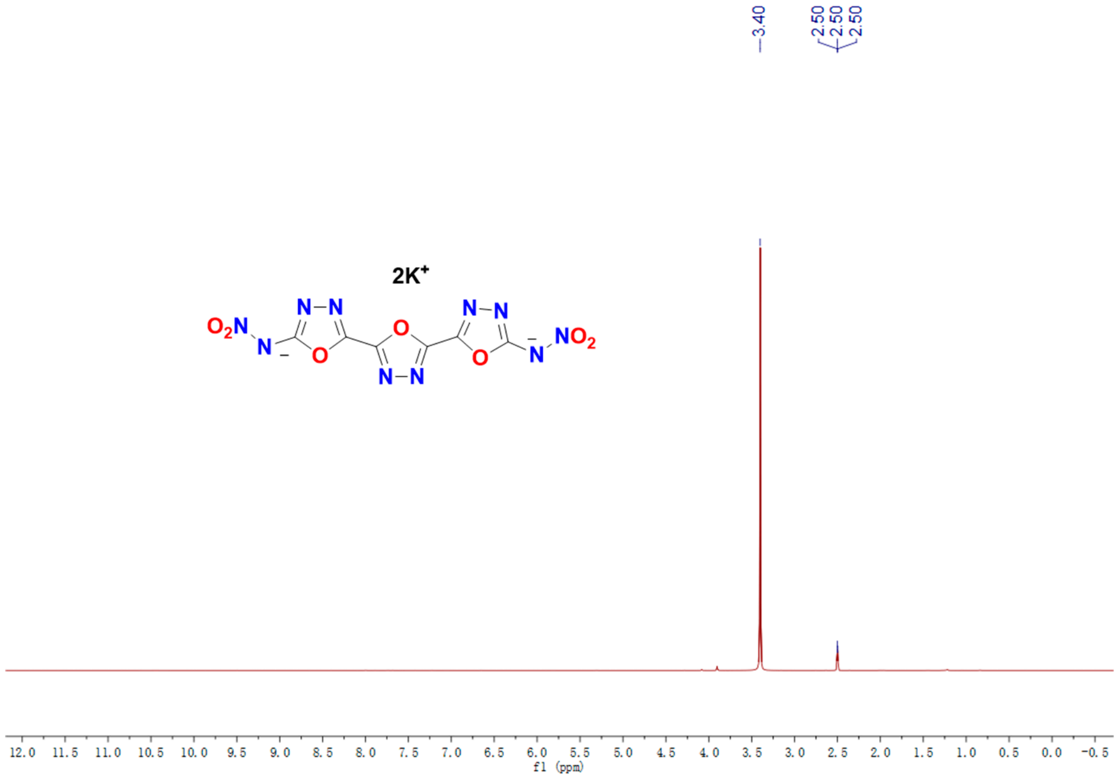

Figure S24. ^{1}H-NMR spectrum of compound **8** in DMSO-d_6.

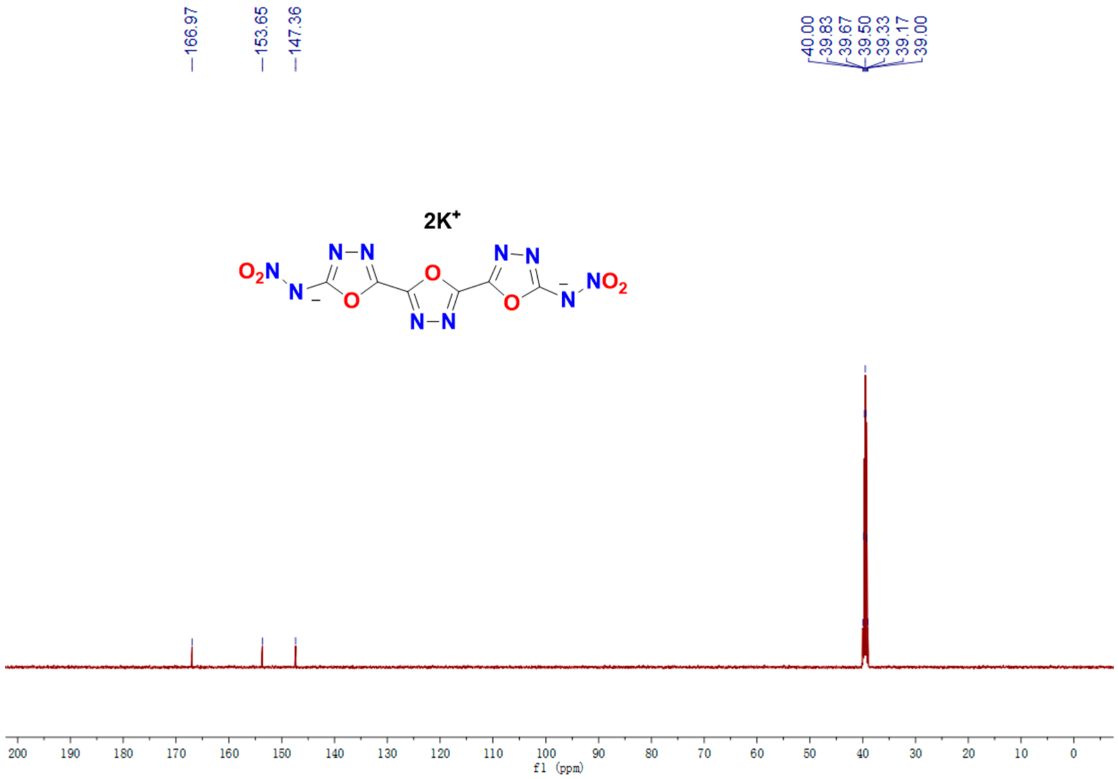

Figure S25. ^{13}C-NMR spectrum of compound **8** in DMSO-d_6.

References

1. Frisch, M.J.; Trucks, G.W.; Schlegel, H.B.; Scuseria, G.E.; Robb, M.A.; Cheeseman, J.R.; Scalmani, G.; Barone, V.; Petersson, G.A.; Nakatsuji, H.; et al. *Gaussian 16*, Revision A.01; Gaussian, Inc.: Wallingford, CT, USA, 2016.
2. Becke, A.D. Density-functional thermochemistry. III. The role of exact exchange. *J. Chem. Phys.* **1993**, *98*, 5648–5652.
3. Hariharan, P.; Pople, J.A. The influence of polarization functions on molecular orbital hydrogenation energies. *Theor. Chim. Acta* **1973**, *28*, 213–222.
4. Stephens, P.J.; Devlin, F.J.; Chabalowski, C.F.; Frisch, M.J. Ab Initio calculation of vibrational absorption and circular dichroism spectra using density functional force fields. *J. Phys. Chem.* **1994**, *98*, 247–257.
5. Mathieu, D. Accurate or fast prediction of solid-state formation enthalpies using standard sublimation enthalpies derived from geometrical fragments. *Ind. Eng. Chem. Res.* **2018**, *57*, 13856–13865.
6. Davis, J.V.; Marrs, F.W.; Cawkwell, M.J.; Manner, V.W. Machine learning models for high explosive crystal density and performance. *Chem. Mater.* **2024**, *36*, 11109–11118.
